# Supplementary material for: Experiment level curation of transcriptional regulatory interactions in neurodevelopment
Source: PLoS Comput Biol. 2021 Oct 19;17(10):e1009484. doi: 10.1371/journal.pcbi.1009484 (PMC8565786; doi:10.1371/journal.pcbi.1009484)
Supplement: S1 Fig — Only TFs with at least one curated target are plotted. Most (212/251) TFs have less than ten targets recorded. (PDF) [file pcbi.1009484.s001.pdf]

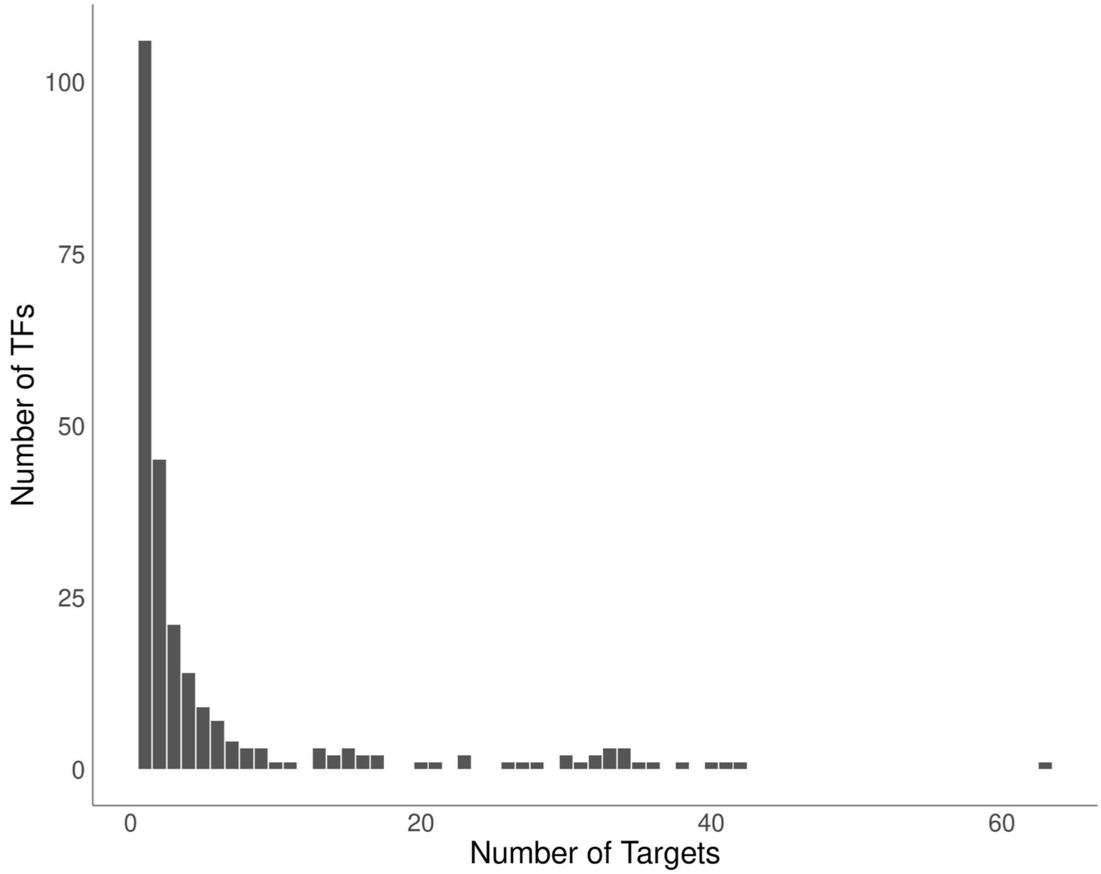

S1 Fig. Distribution of TFs by the number of targets. Only TFs with at least one curated target are plotted. Most (212/251) TFs have less than ten targets recorded.
