# Supplementary material for: Experiment level curation of transcriptional regulatory interactions in neurodevelopment
Source: PLoS Comput Biol. 2021 Oct 19;17(10):e1009484. doi: 10.1371/journal.pcbi.1009484 (PMC8565786; doi:10.1371/journal.pcbi.1009484)
Supplement: S2 Fig — Only targets with at least one recorded TF regulator are included. (PDF) [file pcbi.1009484.s002.pdf]

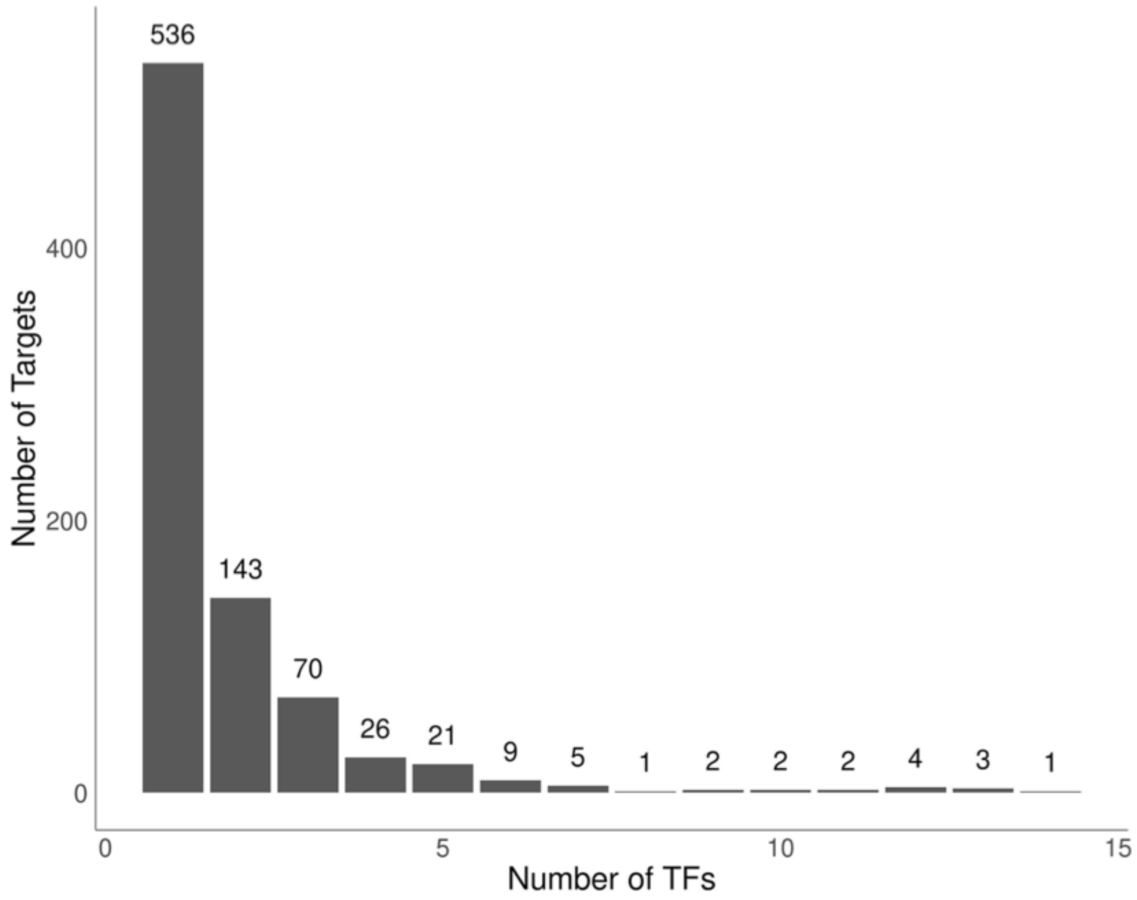

S2 Fig. Distribution of targets by the number of recorded TF regulators. Only targets with at least one recorded TF regulator are included.
