# Supplementary material for: Experiment level curation of transcriptional regulatory interactions in neurodevelopment
Source: PLoS Comput Biol. 2021 Oct 19;17(10):e1009484. doi: 10.1371/journal.pcbi.1009484 (PMC8565786; doi:10.1371/journal.pcbi.1009484)
Supplement: S3 Fig — Target genes that are also neurodevelopmental TFs are indicated by blue dots. Bar colors correspond to cellular contexts indicated in the color legend. There are eight targets with more than 10 recorded TF regulators (denoted by the horizontal dotted line). (PDF) [file pcbi.1009484.s003.pdf]

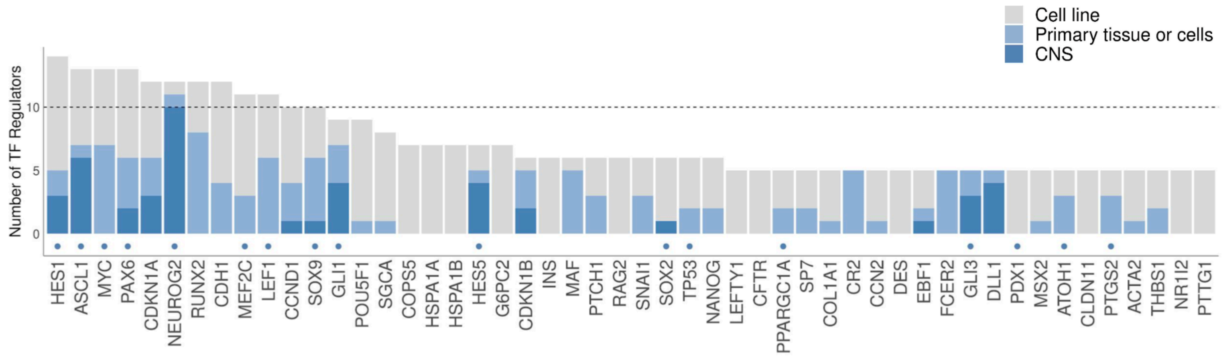

S3 Fig. Number of TF regulators per target for the top 50 target genes. Target genes that are also neurodevelopmental TFs are indicated by blue dots. Bar colors correspond to cellular contexts indicated in the color legend. There are eight targets with more than 10 recorded TF regulators (denoted by the horizontal dotted line).
