# Supplementary material for: Experiment level curation of transcriptional regulatory interactions in neurodevelopment
Source: PLoS Comput Biol. 2021 Oct 19;17(10):e1009484. doi: 10.1371/journal.pcbi.1009484 (PMC8565786; doi:10.1371/journal.pcbi.1009484)
Supplement: S4 Fig — Colors correspond to cellular contexts as indicated in the legend. Top left: Breakdown of experiments by species. The “mixed” column refers to experiments where the TF and the target genes originated from different species, which is possible in TF overexpression experiments. Top right: Breakdown of experiments by context type. Bottom left: Breakdown of experiments by the mode of TF perturbation. “Knock Out” refers perturbations at the genetic level including naturally occurring mutations. Further breakdown into heterozygous or homozygous knock outs are provided in S5 Data. “Knock Down” refers to transcript level perturbation by RNA interference. Bottom right: Breakdown of experiments by the effect of TF perturbation in primary tissues or cells. “Constitutive” perturbations are present throughout development versus “induced” perturbations are triggered closer to the time of assay. (PDF) [file pcbi.1009484.s004.pdf]

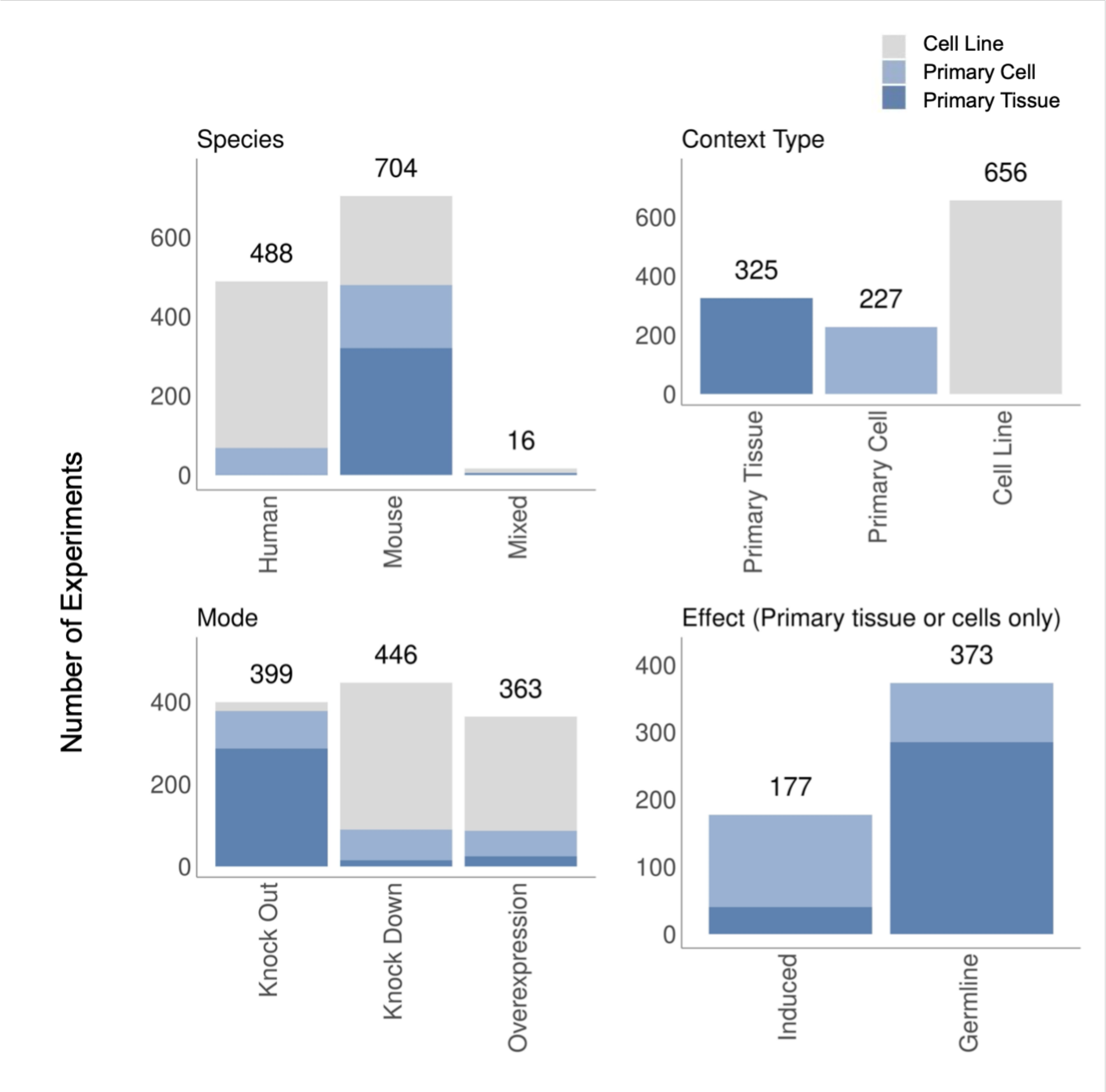

S4 Fig. Details of TF perturbation experiments. Colors correspond to cellular contexts as indicated in the legend. Top left: Breakdown of experiments by species. The “mixed” column refers to experiments where the TF and the target genes originated from different species, which is possible in TF overexpression experiments. Top right: Breakdown of experiments by context type. Bottom left: Breakdown of experiments by the mode of TF perturbation. “Knock Out” refers perturbations at the genetic level

including naturally occurring mutations. Further breakdown into heterozygous or homozygous knock outs are provided in S5 Data. “Knock Down” refers to transcript level perturbation by RNA interference. Bottom right: Breakdown of experiments by the effect of TF perturbation in primary tissues or cells. “Constitutive” perturbations are present throughout development versus “induced” perturbations are triggered closer to the time of assay.
