# Supplementary material for: Experiment level curation of transcriptional regulatory interactions in neurodevelopment
Source: PLoS Comput Biol. 2021 Oct 19;17(10):e1009484. doi: 10.1371/journal.pcbi.1009484 (PMC8565786; doi:10.1371/journal.pcbi.1009484)
Supplement: S5 Fig — Colors correspond to cellular contexts as indicated in the color legend. Top left: Breakdown of experiments by species. The “mixed” column refers to experiments where the TF and the target genes originated from different species, which is possible in EMSA experiments. Top right: Breakdown of experiments by context type. All EMSA experiments were classified as “in-vitro” for context type. Bottom left: Breakdown of experiments by method. Bottom right: Breakdown of experiments by source of the TF protein in EMSA experiments. Forty-eight EMSA experiments used endogenous TF proteins sourced from primary tissues or cells. In most cases (292), TF proteins were sourced from cell lines following TF transfection. (PDF) [file pcbi.1009484.s005.pdf]

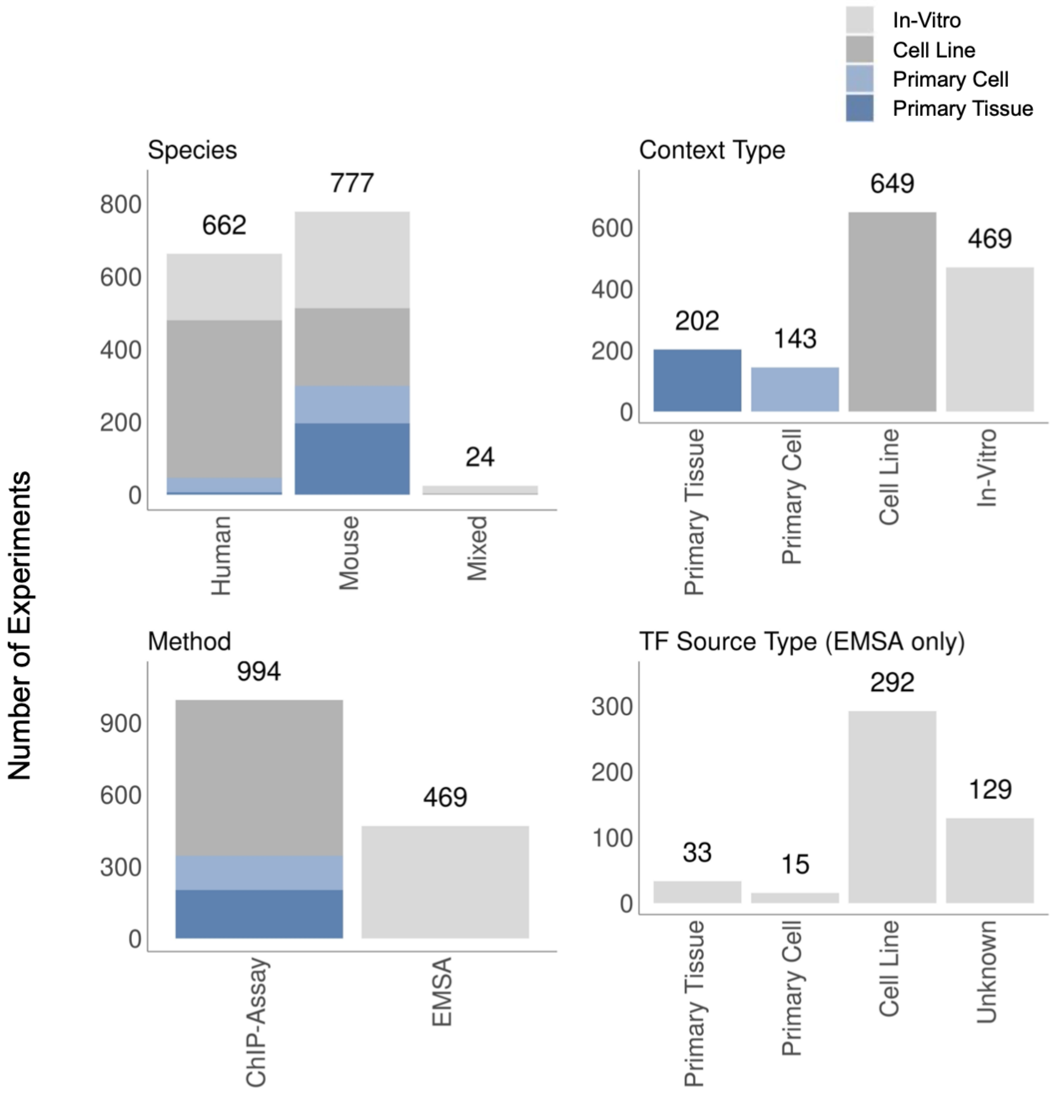

S5 Fig. Details of TF-DNA binding experiments. Colors correspond to cellular contexts as indicated in the color legend. Top left: Breakdown of experiments by species. The “mixed” column refers to experiments where the TF and the target genes originated from different species, which is possible in EMSA experiments. Top right: Breakdown of experiments by context type. All EMSA experiments were classified as “in-vitro” for context type. Bottom left: Breakdown of experiments by method. Bottom right:

Breakdown of experiments by source of the TF protein in EMSA experiments. Forty-eight EMSA experiments used endogenous TF proteins sourced from primary tissues or cells. In most cases (292), TF proteins were sourced from cell lines following TF transfection.
