# Supplementary material for: Experiment level curation of transcriptional regulatory interactions in neurodevelopment
Source: PLoS Comput Biol. 2021 Oct 19;17(10):e1009484. doi: 10.1371/journal.pcbi.1009484 (PMC8565786; doi:10.1371/journal.pcbi.1009484)
Supplement: S6 Fig — Colors correspond to cellular contexts as indicated in the color legend. Top left: Breakdown of experiments by species. The “mixed” column refers to experiments where the TF and the target genes originated from different species. Top right: Breakdown of experiments by context type. Few TF-reporter experiments were performed using primary tissues or cells. Bottom left: Breakdown of TF-reporter experiments that did or did not investigate the effect of mutating the corresponding cRE sequence. Bottom right: Breakdown of whether the effect of the mutation on the TF-DNA interaction was confirmed experimentally by EMSA. (PDF) [file pcbi.1009484.s006.pdf]

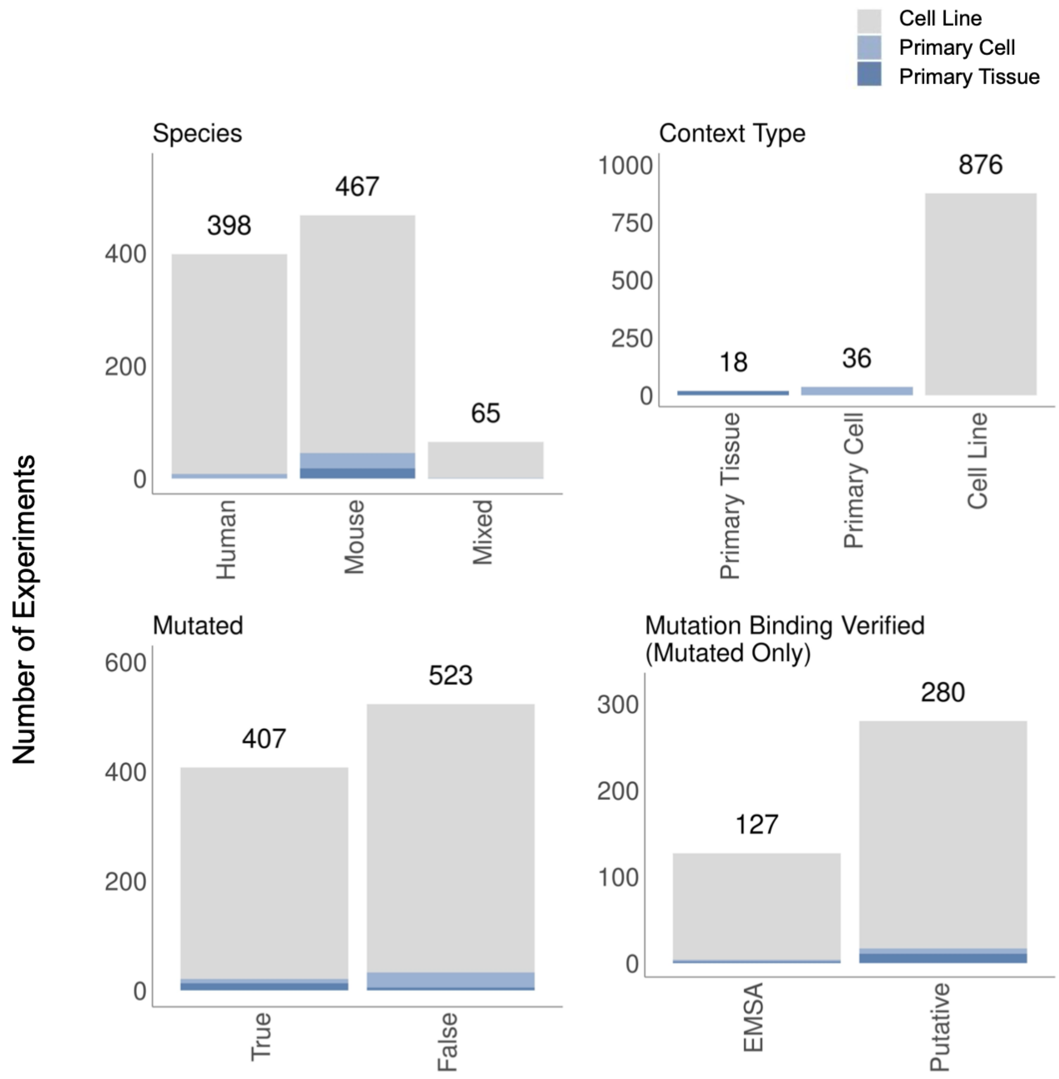

S6 Fig. Details of TF-reporter experiments. Colors correspond to cellular contexts as indicated in the color legend. Top left: Breakdown of experiments by species. The “mixed” column refers to experiments where the TF and the target genes originated from different species. Top right: Breakdown of experiments by context type. Few TF-reporter experiments were performed using primary tissues or cells. Bottom left: Breakdown of TF-reporter experiments that did or did not investigate the effect of

mutating the corresponding cRE sequence. Bottom right: Breakdown of whether the effect of the mutation on the TF-DNA interaction was confirmed experimentally by EMSA.
