# Supplementary material for: Experiment level curation of transcriptional regulatory interactions in neurodevelopment
Source: PLoS Comput Biol. 2021 Oct 19;17(10):e1009484. doi: 10.1371/journal.pcbi.1009484 (PMC8565786; doi:10.1371/journal.pcbi.1009484)
Supplement: S7 Fig — DTRI counts are plotted on the left and experiment counts are plotted on the right. Additional columns are shown for experiments performed using embryonic tissue or cells. DTRIs and experiments involving neurodevelopmental TF regulators are highlighted in blue. (PDF) [file pcbi.1009484.s007.pdf]

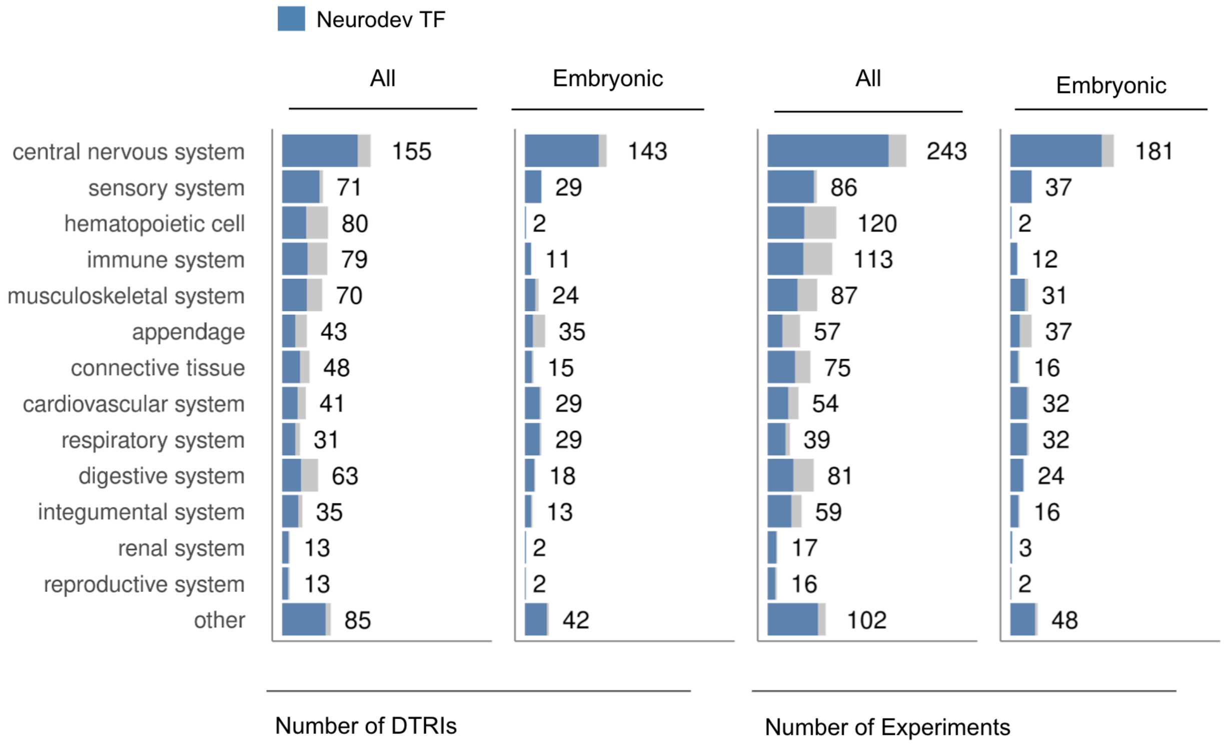

S7 Fig. Breakdown of experiments performed using primary tissues or cells across anatomical systems. DTRI counts are plotted on the left and experiment counts are plotted on the right. Additional columns are shown for experiments performed using embryonic tissue or cells. DTRIs and experiments involving neurodevelopmental TF regulators are highlighted in blue.
