# Supplementary material for: Experiment level curation of transcriptional regulatory interactions in neurodevelopment
Source: PLoS Comput Biol. 2021 Oct 19;17(10):e1009484. doi: 10.1371/journal.pcbi.1009484 (PMC8565786; doi:10.1371/journal.pcbi.1009484)
Supplement: S8 Fig — DTRI counts are plotted on the left and experiment counts are plotted on the right. DTRIs involving neurodevelopmental TF regulators are highlighted in blue. (PDF) [file pcbi.1009484.s008.pdf]

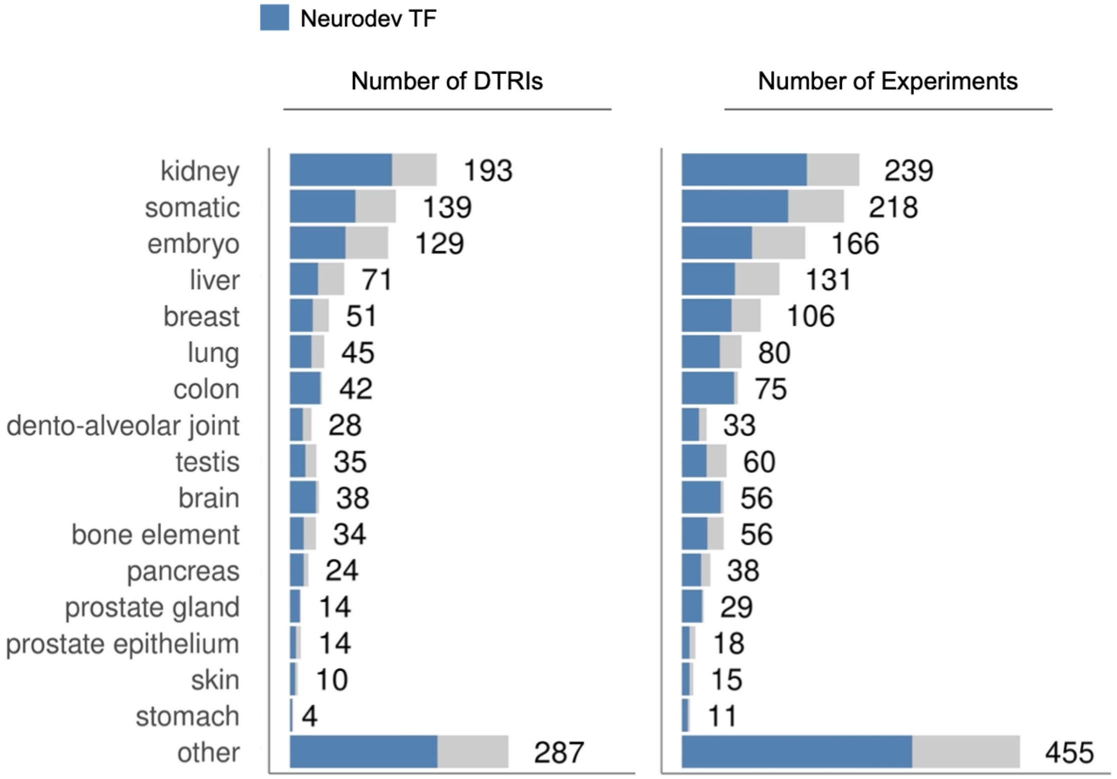

S8 Fig. Breakdown of experiments performed using cell lines across broad cell type categories. DTRI counts are plotted on the left and experiment counts are plotted on the right. DTRIs involving neurodevelopmental TF regulators are highlighted in blue.
