# Supplementary material for: Experiment level curation of transcriptional regulatory interactions in neurodevelopment
Source: PLoS Comput Biol. 2021 Oct 19;17(10):e1009484. doi: 10.1371/journal.pcbi.1009484 (PMC8565786; doi:10.1371/journal.pcbi.1009484)
Supplement: S9 Fig — Overlap values are reported as fractions of the “target” resource (x-axis). Only values of 0.05 or higher are printed. For example, TRRUST contains 0.4 of the DTRIs recorded in our curation whereas we captured less than 0.05 of the DTRIs in TRRUST. External resources are ordered by the number of recorded DTRIs. (PDF) [file pcbi.1009484.s009.pdf]

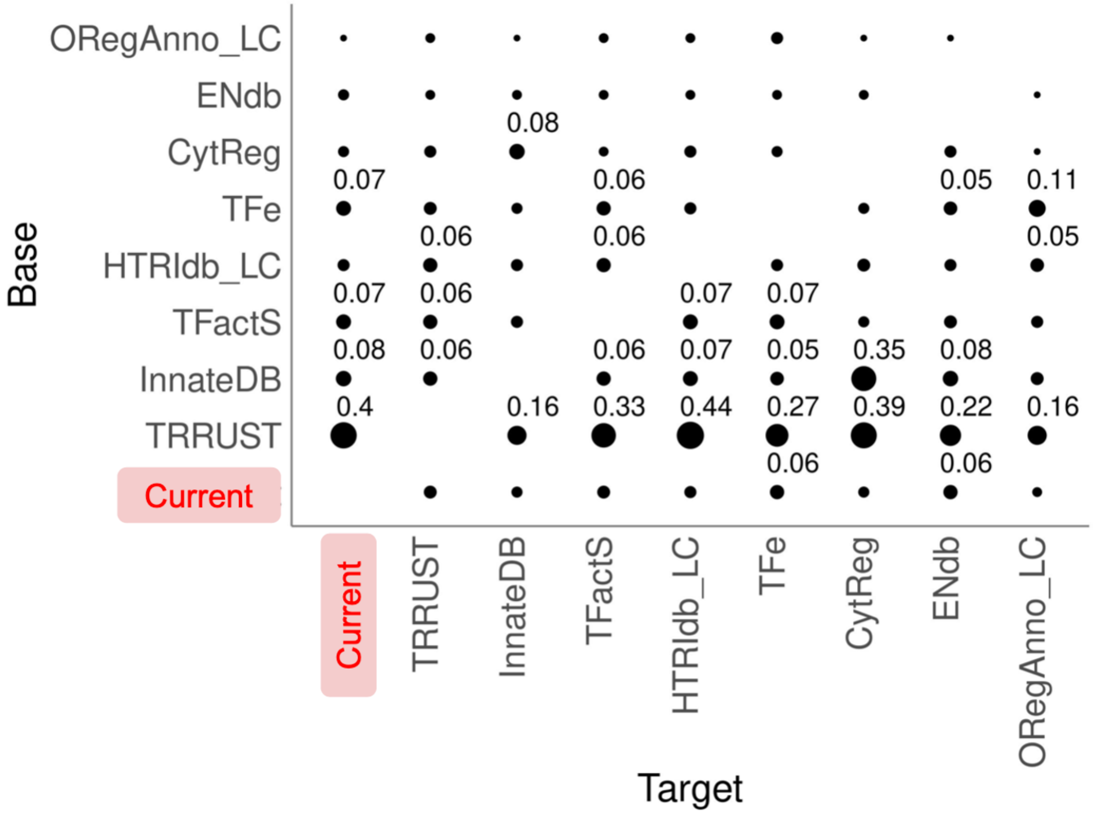

S9 Fig. Pairwise overlap of DTRIs among the different data resources. Overlap values are reported as fractions of the "target" resource (x-axis). Only values of 0.05 or higher are printed. For example, TRRUST contains 0.4 of the DTRIs recorded in our curation whereas we captured less than 0.05 of the DTRIs in TRRUST. External resources are ordered by the number of recorded DTRIs.
