# Supplementary material for: Experiment level curation of transcriptional regulatory interactions in neurodevelopment
Source: PLoS Comput Biol. 2021 Oct 19;17(10):e1009484. doi: 10.1371/journal.pcbi.1009484 (PMC8565786; doi:10.1371/journal.pcbi.1009484)
Supplement: S10 Fig — Only papers with at least one curated DTRI are included. The majority of papers report only a single DTRI. (PDF) [file pcbi.1009484.s010.pdf]

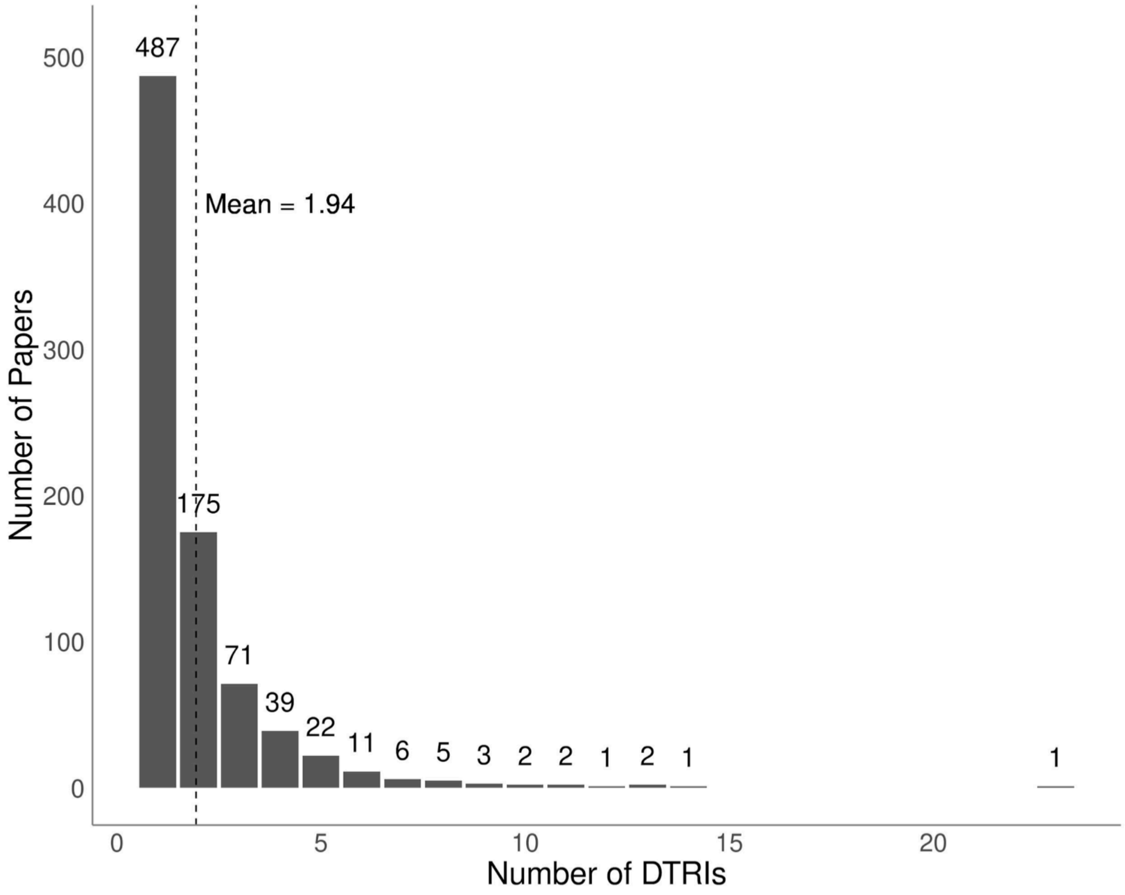

S10 Fig. Distribution of papers by the number of DTRIs reported. Only papers with at least one curated DTRI are included. The majority of papers report only a single DTRI.
