# Supplementary material for: Experiment level curation of transcriptional regulatory interactions in neurodevelopment
Source: PLoS Comput Biol. 2021 Oct 19;17(10):e1009484. doi: 10.1371/journal.pcbi.1009484 (PMC8565786; doi:10.1371/journal.pcbi.1009484)
Supplement: S11 Fig — For “TFBS Position” (y-axis), “Proximal” refers to promoters or cREs that are within 2 kb of the target TSS as reported in the original publication. The “Distal” category includes cREs further than 2 kb upstream or downstream from the target TSS. “Both” includes DTRIs with both proximal and distal regulatory elements. For “Mode of Regulation”, “Activation” refers to TF perturbation or TF-reporter assays where the direction of change in target gene expression is the same as direction of the corresponding TF perturbation. Similarly, “Repression” includes results where expression of the target gene changes in the opposite direction. “Both” refers to DTRIs with alternative results from different experiments. (PDF) [file pcbi.1009484.s011.pdf]

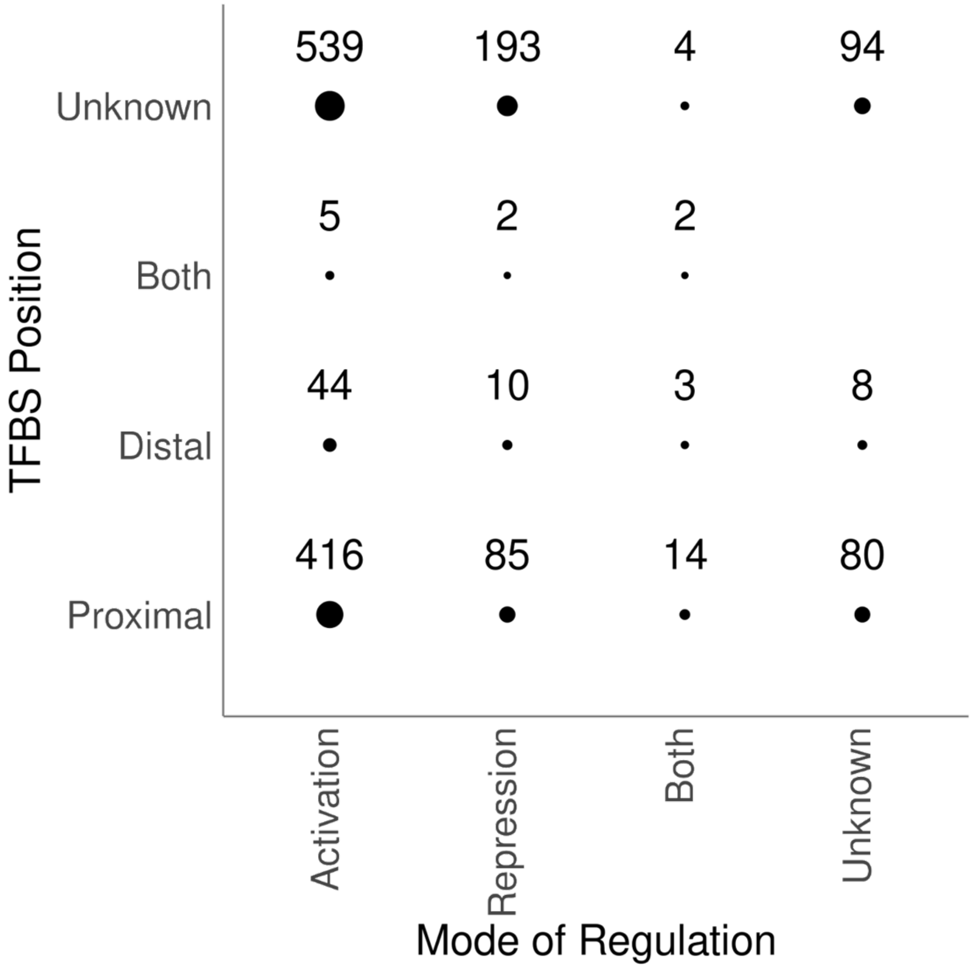

S11 Fig. Breakdown of DTRIs by mode of regulation and TFBS position. For “TFBS Position” (y-axis), “Proximal” refers to promoters or cREs that are within 2 kb of the target TSS as reported in the original publication. The “Distal” category includes cREs further than 2 kb upstream or downstream from the target TSS. “Both” includes DTRIs with both proximal and distal regulatory elements. For “Mode of Regulation”, “Activation” refers to TF perturbation or TF-reporter assays where the direction of change in target gene expression is the same as direction of the corresponding TF

perturbation. Similarly, “Repression” includes results where expression of the target gene changes in the opposite direction. “Both” refers to DTRIs with alternative results from different experiments.
