# Supplementary material for: Experiment level curation of transcriptional regulatory interactions in neurodevelopment
Source: PLoS Comput Biol. 2021 Oct 19;17(10):e1009484. doi: 10.1371/journal.pcbi.1009484 (PMC8565786; doi:10.1371/journal.pcbi.1009484)
Supplement: S12 Fig — Colors correspond to TFBS position annotations. Dotted line indicates 10 targets on the y-axis for reference. (PDF) [file pcbi.1009484.s012.pdf]

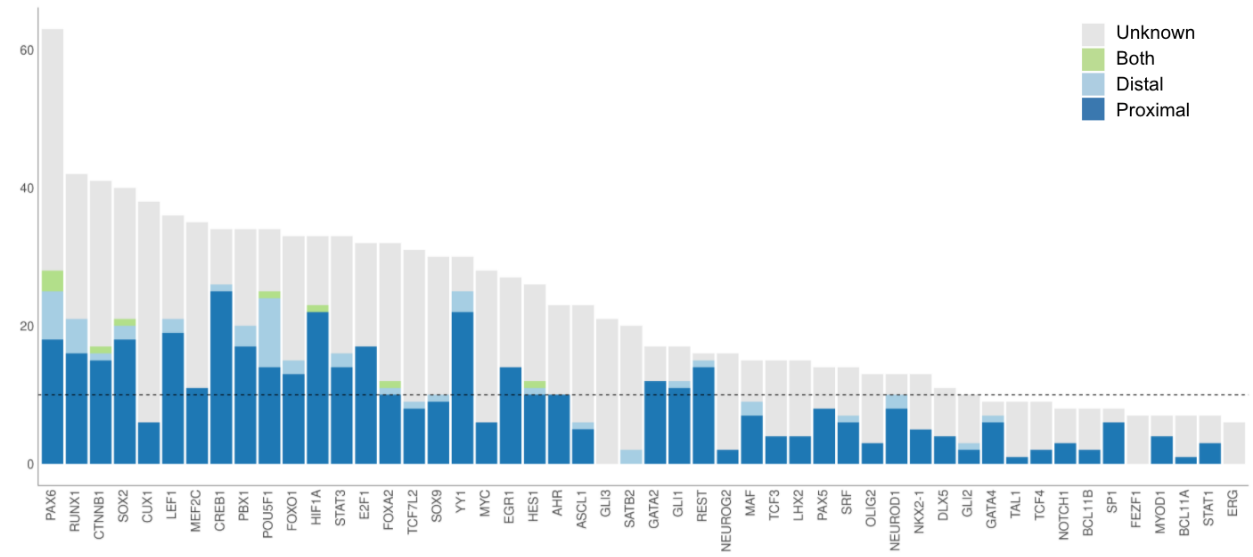

S12 Fig. TFBS Position of DTRIs per TF for the top 50 TFs. Colors correspond to TFBS position annotations. Dotted line indicates 10 targets on the y-axis for reference.
