# Supplementary material for: Experiment level curation of transcriptional regulatory interactions in neurodevelopment
Source: PLoS Comput Biol. 2021 Oct 19;17(10):e1009484. doi: 10.1371/journal.pcbi.1009484 (PMC8565786; doi:10.1371/journal.pcbi.1009484)
Supplement: S13 Fig — TFBS coordinates are given relative to the TSS of the target gene. Only the coordinates of the ends closer to the target genes’ TSS were recorded. Dotted lines (+/-2000) indicate the threshold for calling proximal or distal TFBSs. Records with coordinates above or below +/-10000 are not included in this figure. (PDF) [file pcbi.1009484.s013.pdf]

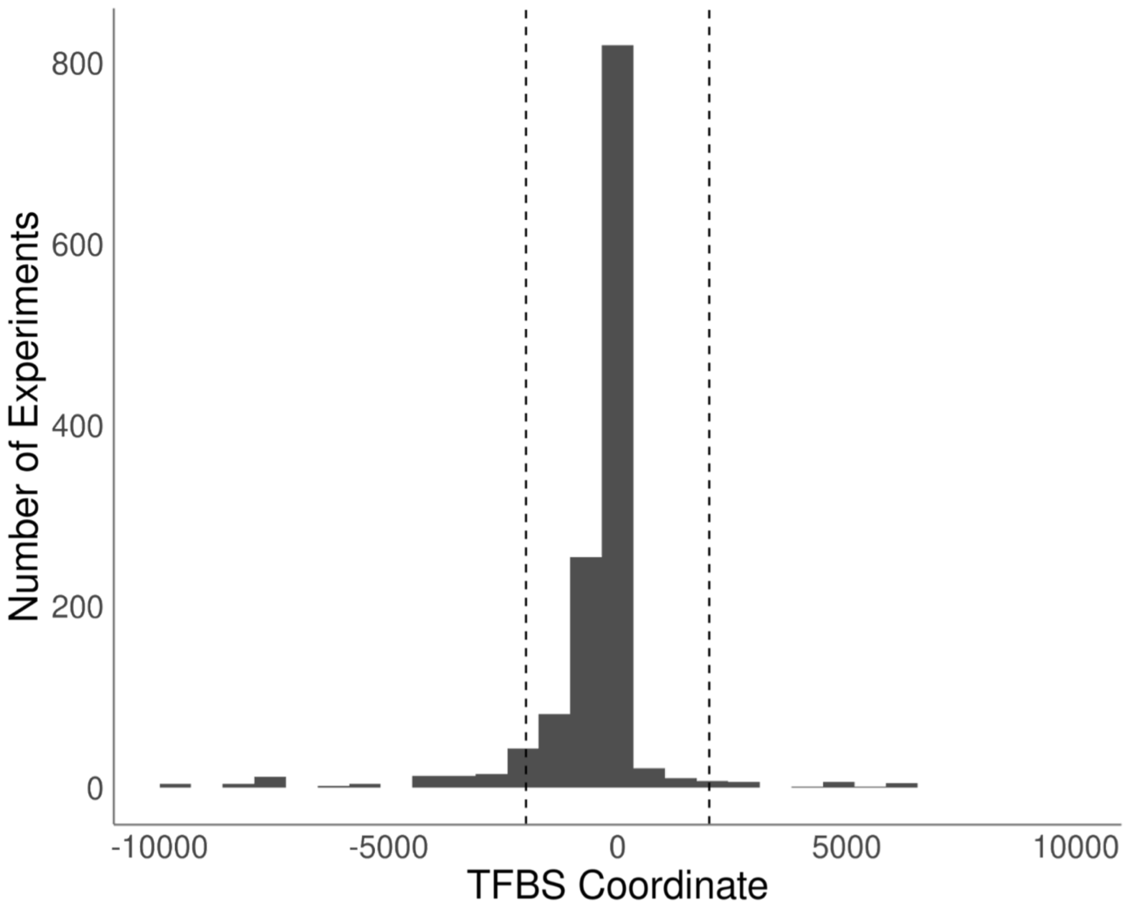

S13 Fig. Distribution of TFBS coordinates across experiments. TFBS coordinates are given relative to the TSS of the target gene. Only the coordinates of the ends closer to the target genes' TSS were recorded. Dotted lines (+/-2000) indicate the threshold for calling proximal or distal TFBSs. Records with coordinates above or below +/-10000 are not included in this figure.
