# Supplementary material for: Experiment level curation of transcriptional regulatory interactions in neurodevelopment
Source: PLoS Comput Biol. 2021 Oct 19;17(10):e1009484. doi: 10.1371/journal.pcbi.1009484 (PMC8565786; doi:10.1371/journal.pcbi.1009484)
Supplement: S14 Fig — Colors correspond to the mode of regulation annotations. The dotted line indicates 10 on the y-axis for reference. Most TFs appear to be primarily activators whereas a small number of TFs such as HES1, GLI3, and REST repress the majority of their recorded targets. (PDF) [file pcbi.1009484.s014.pdf]

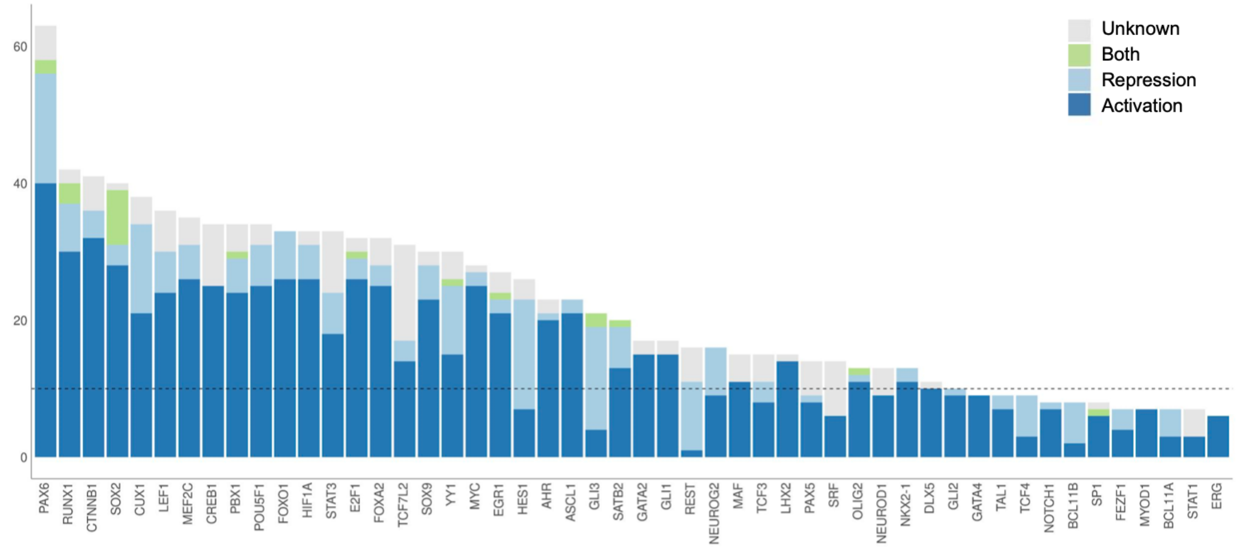

S14 Fig. Mode of regulation of DTRIs per TF for the top 50 TFs. Colors correspond to the mode of regulation annotations. The dotted line indicates 10 on the y-axis for reference. Most TFs appear to be primarily activators whereas a small number of TFs such as HES1, GLI3, and REST repress the majority of their recorded targets.
