# Supplementary material for: Experiment level curation of transcriptional regulatory interactions in neurodevelopment
Source: PLoS Comput Biol. 2021 Oct 19;17(10):e1009484. doi: 10.1371/journal.pcbi.1009484 (PMC8565786; doi:10.1371/journal.pcbi.1009484)
Supplement: S15 Fig — AUROC and the corresponding p-value (Mann-Whitney U Test) are displayed in the panel. (PDF) [file pcbi.1009484.s015.pdf]

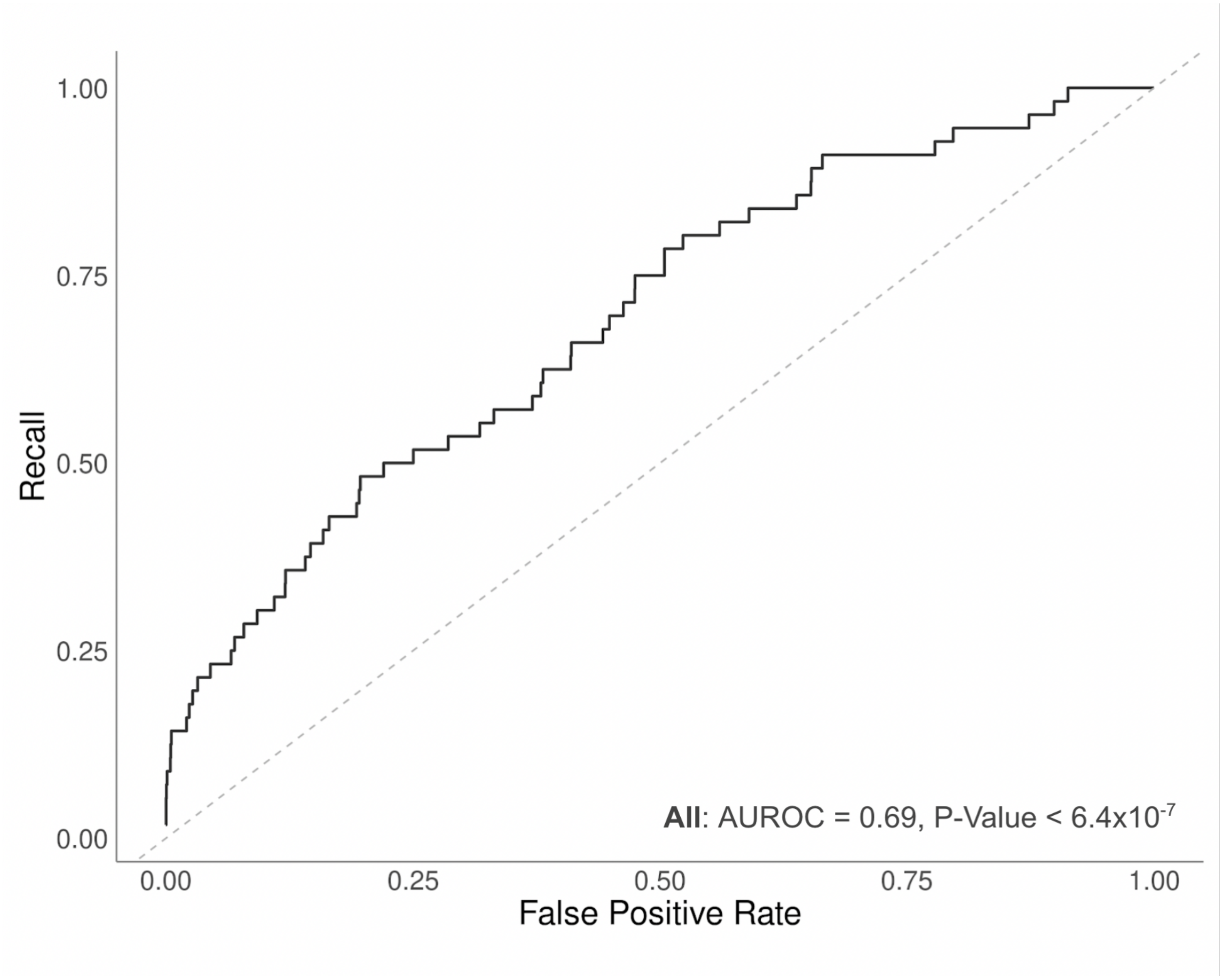

S15 Fig. Enrichment of all curated PAX6/Pax6 targets among differentially expressed genes in Walcher et al., 2013 [1]. AUROC and the corresponding p-value (Mann-Whitney U Test) are displayed in the panel.

References

1. Walcher T, Xie Q, Sun J, Irmler M, Beckers J, Öztürk T, et al. Functional dissection of the paired domain of Pax6 reveals molecular mechanisms of coordinating neurogenesis and proliferation. Development. 2013;140: 1123–1136. doi:10.1242/dev.082875
