# Supplementary material for: Experiment level curation of transcriptional regulatory interactions in neurodevelopment
Source: PLoS Comput Biol. 2021 Oct 19;17(10):e1009484. doi: 10.1371/journal.pcbi.1009484 (PMC8565786; doi:10.1371/journal.pcbi.1009484)
Supplement: S16 Fig — Targets are classified based on the reported cellular contexts. AUROCs and the corresponding p-values (Mann-Whitney U Test) are displayed in the panel. Color coding corresponds to categories of targets. Targets with experimental validation in primary CNS tissues are most enriched in the perturbation screen. No statistically significant enrichment is observed for targets tested in tissues or cell types other than the CNS or the eye. (PDF) [file pcbi.1009484.s016.pdf]

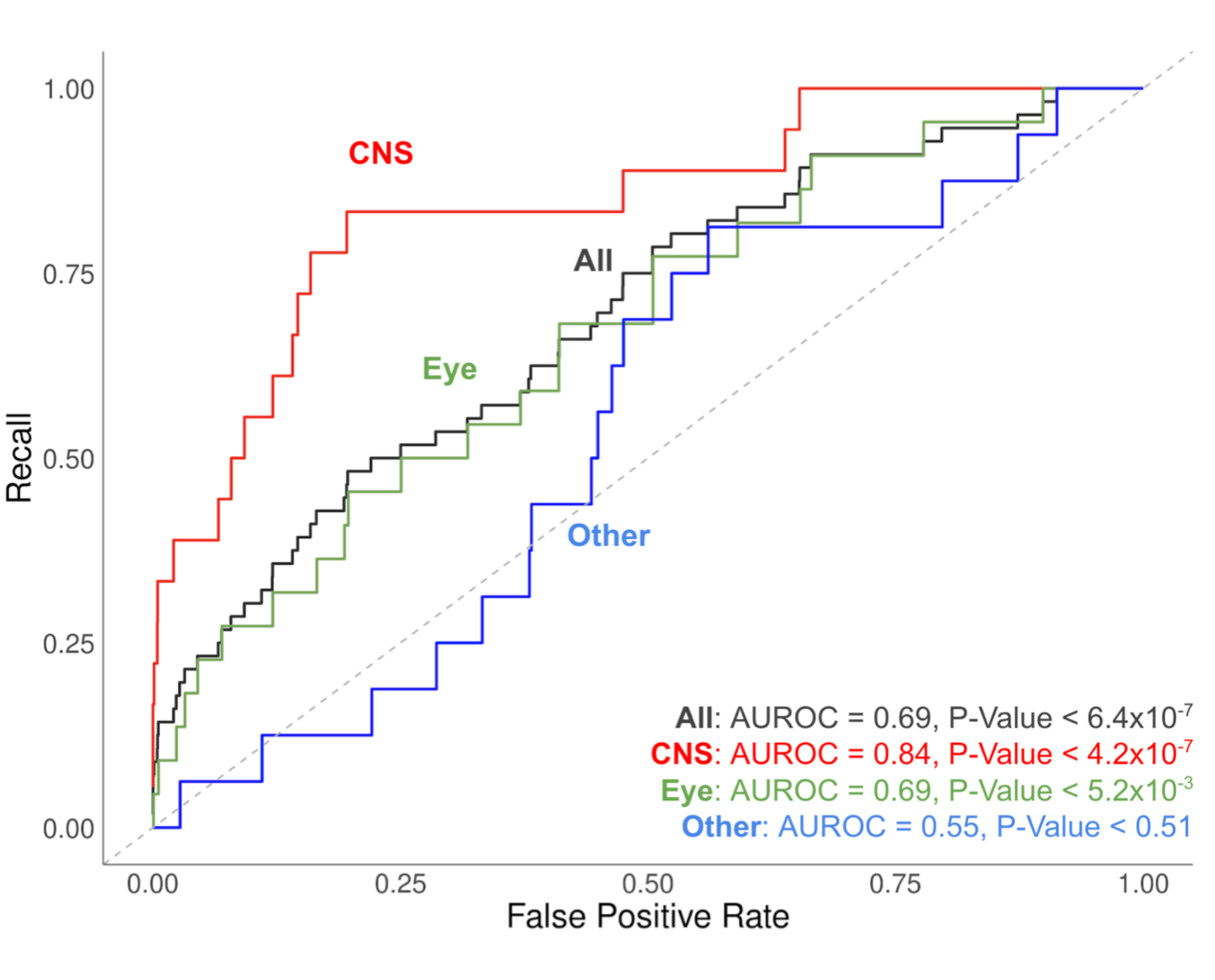

S16 Fig. Enrichment of curated PAX6/Pax6 targets among differentially expressed genes in Walcher et al., 2013 [1] by tissue types. Targets are classified based on the reported cellular contexts. AUROCs and the corresponding p-values (Mann-Whitney U Test) are displayed in the panel. Color coding corresponds to categories of targets. Targets with experimental validation in primary CNS tissues are most enriched in the perturbation screen. No statistically significant enrichment is observed for targets tested in tissues or cell types other than the CNS or the eye.

References

1. Walcher T, Xie Q, Sun J, Irmler M, Beckers J, Öztürk T, et al. Functional dissection of the paired domain of Pax6 reveals molecular mechanisms of coordinating neurogenesis and proliferation. Development. 2013;140: 1123–1136. doi:10.1242/dev.082875
