# Supplementary material for: Experiment level curation of transcriptional regulatory interactions in neurodevelopment
Source: PLoS Comput Biol. 2021 Oct 19;17(10):e1009484. doi: 10.1371/journal.pcbi.1009484 (PMC8565786; doi:10.1371/journal.pcbi.1009484)
Supplement: S17 Fig — Confidence intervals (95th percentile) were derived by bootstrapping 1000 random samples from each category. Statistically significant differences were observed between the CNS versus “other”. A large difference was also observed between targets with multiple versus a single type of low-throughput experimental evidence, though it did not pass the threshold for statistical significance. Dotted line of AUROC = 0.5 indicates random expectation. (PDF) [file pcbi.1009484.s017.pdf]

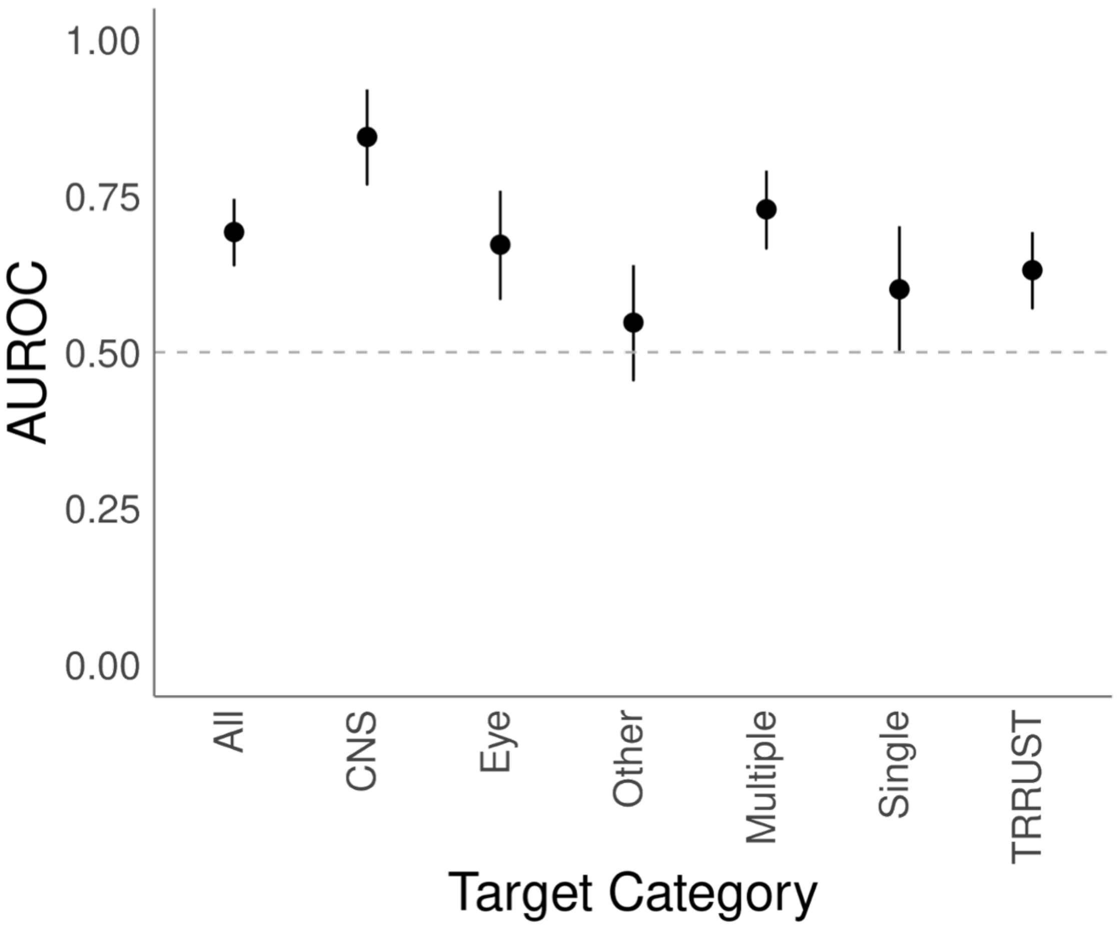

S17 Fig. Enrichment levels (measured in AUROC) for the different categories of curated PAX6/Pax6 targets among differentially expressed in genes in Walcher et al., 2013 [1]. Confidence intervals (95th percentile) were derived by bootstrapping 1000 random samples from each category. Statistically significant differences were observed between the CNS versus “other”. A large difference was also observed between targets with multiple versus a single type of low-throughput experimental evidence, though it did not pass the threshold for statistical significance. Dotted line of AUROC = 0.5 indicates random expectation.

References

1. Walcher T, Xie Q, Sun J, Irmler M, Beckers J, Öztürk T, et al. Functional dissection of the paired domain of Pax6 reveals molecular mechanisms of coordinating neurogenesis and proliferation. Development. 2013;140: 1123–1136. doi:10.1242/dev.082875
