# Supplementary material for: Experiment level curation of transcriptional regulatory interactions in neurodevelopment
Source: PLoS Comput Biol. 2021 Oct 19;17(10):e1009484. doi: 10.1371/journal.pcbi.1009484 (PMC8565786; doi:10.1371/journal.pcbi.1009484)
Supplement: S18 Fig — AUROCs and the corresponding p-values (Mann-Whitney U Test) are displayed in the panel. Color coding corresponds to categories of targets. No statistically significant enrichment is observed for targets tested only in a single type of experiment. (PDF) [file pcbi.1009484.s018.pdf]

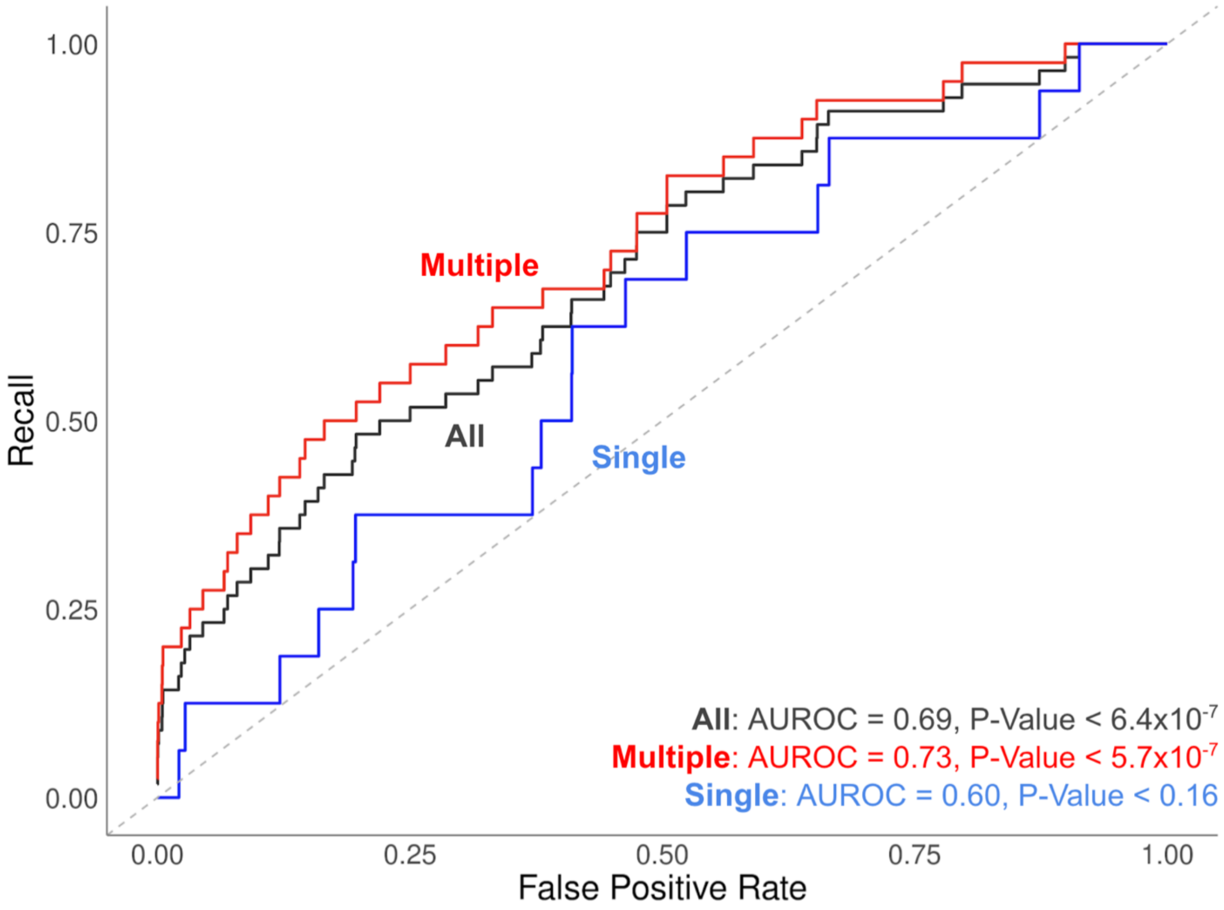

S18 Fig. Enrichment of curated PAX6/Pax6 targets among differentially expressed genes in Walcher et al., 2013 [1] by number of experiment types. AUROCs and the corresponding p-values (Mann-Whitney U Test) are displayed in the panel. Color coding corresponds to categories of targets. No statistically significant enrichment is observed for targets tested only in a single type of experiment.

References

1. Walcher T, Xie Q, Sun J, Irmler M, Beckers J, Öztürk T, et al. Functional dissection of the paired domain of Pax6 reveals molecular mechanisms of coordinating neurogenesis and proliferation. Development. 2013;140: 1123–1136. doi:10.1242/dev.082875
