# Supplementary material for: Experiment level curation of transcriptional regulatory interactions in neurodevelopment
Source: PLoS Comput Biol. 2021 Oct 19;17(10):e1009484. doi: 10.1371/journal.pcbi.1009484 (PMC8565786; doi:10.1371/journal.pcbi.1009484)
Supplement: S19 Fig — Confidence intervals (95th percentile) were derived by bootstrapping 1000 random samples from each category. The general trend from the comparison with Walcher et al., 2013 [36] was replicated (S17 Fig) though the differences did not pass the threshold for statistical significance here. Dotted line of AUROC = 0.5 indicates random expectation. (PDF) [file pcbi.1009484.s019.pdf]

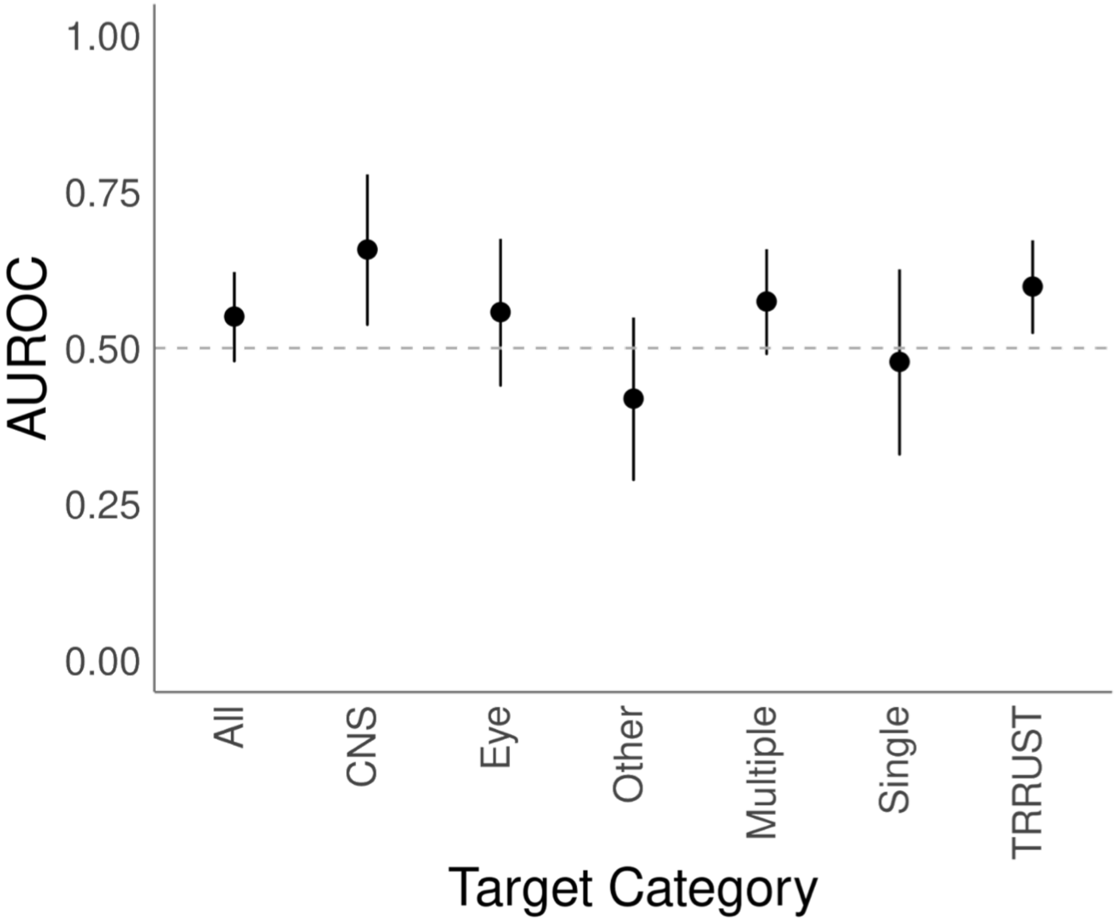

S19 Fig. Enrichment levels (measured in AUROC) for the different categories of curated PAX6/Pax6 targets among differentially expressed in genes in Narayanan et al., 2018 [1]. Confidence intervals (95th percentile) were derived by bootstrapping 1000 random samples from each category. The general trend from the comparison with Walcher et al., 2013 [2] was replicated (S17 Fig) though the differences did not pass the threshold for statistical significance here. Dotted line of AUROC = 0.5 indicates random expectation.

References

1. Narayanan R, Pham L, Kerimoglu C, Watanabe T, Castro Hernandez R, Sokpor G, et al. Chromatin Remodeling BAF155 Subunit Regulates the Genesis of Basal Progenitors in Developing Cortex. iScience. 2018;4: 109–126. doi:10.1016/j.isci.2018.05.014

2. Walcher T, Xie Q, Sun J, Irmler M, Beckers J, Öztürk T, et al. Functional dissection of the paired domain of Pax6 reveals molecular mechanisms of coordinating neurogenesis and proliferation. Development. 2013;140: 1123–1136. doi:10.1242/dev.082875
