# Supplementary material for: Experiment level curation of transcriptional regulatory interactions in neurodevelopment
Source: PLoS Comput Biol. 2021 Oct 19;17(10):e1009484. doi: 10.1371/journal.pcbi.1009484 (PMC8565786; doi:10.1371/journal.pcbi.1009484)
Supplement: S20 Fig — AUROCs and the corresponding p-values (Mann-Whitney U Test) are displayed in the panel. Color coding corresponds to categories of targets. Only the targets experimentally verified in the CNS show statistically significant enrichment. (PDF) [file pcbi.1009484.s020.pdf]

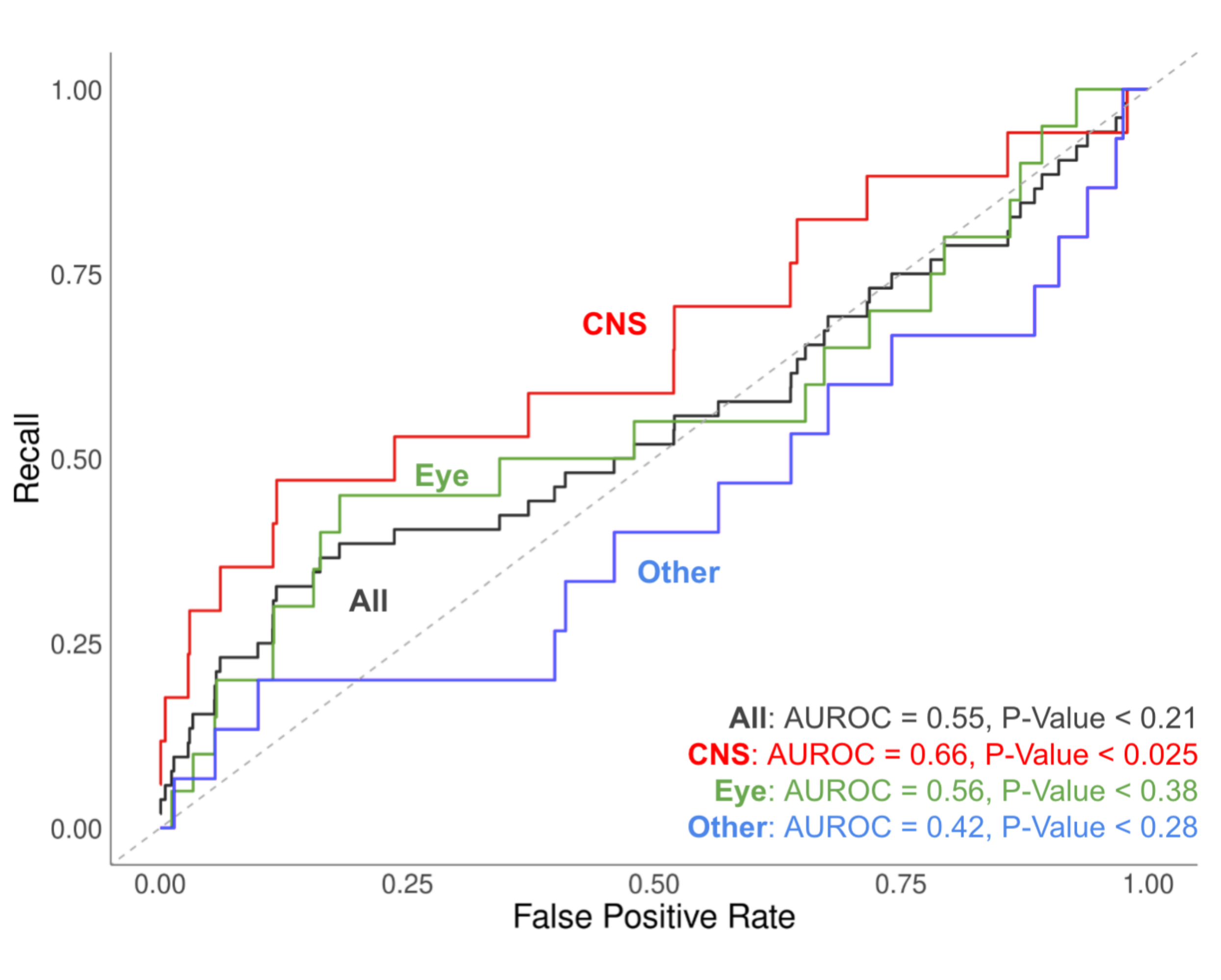

S20 Fig. Enrichment of curated PAX6/Pax6 targets among differentially expressed genes in Narayanan et al., 2018 [1] by tissue types. AUROCs and the corresponding p-values (Mann-Whitney U Test) are displayed in the panel. Color coding corresponds to categories of targets. Only the targets experimentally verified in the CNS show statistically significant enrichment.

References

1. Narayanan R, Pham L, Kerimoglu C, Watanabe T, Castro Hernandez R, Sokpor G, et al. Chromatin Remodeling BAF155 Subunit Regulates the Genesis of Basal Progenitors in Developing Cortex. iScience. 2018;4: 109–126. doi:10.1016/j.isci.2018.05.014
