# Supplementary material for: Experiment level curation of transcriptional regulatory interactions in neurodevelopment
Source: PLoS Comput Biol. 2021 Oct 19;17(10):e1009484. doi: 10.1371/journal.pcbi.1009484 (PMC8565786; doi:10.1371/journal.pcbi.1009484)
Supplement: S21 Fig — Each point is a high-throughput data set. Stars denote enrichment in the consensus ranking across all datasets for each TF. TFs are ordered by decreasing AUROCs in the consensus ranking. For eight of the 10 TFs, the consensus rankings are more enriched for the curated targets than the median across all datasets. Colors indicate statistical significance. For four TFs, the consensus rankings are significantly enriched for the curated targets. (PDF) [file pcbi.1009484.s021.pdf]

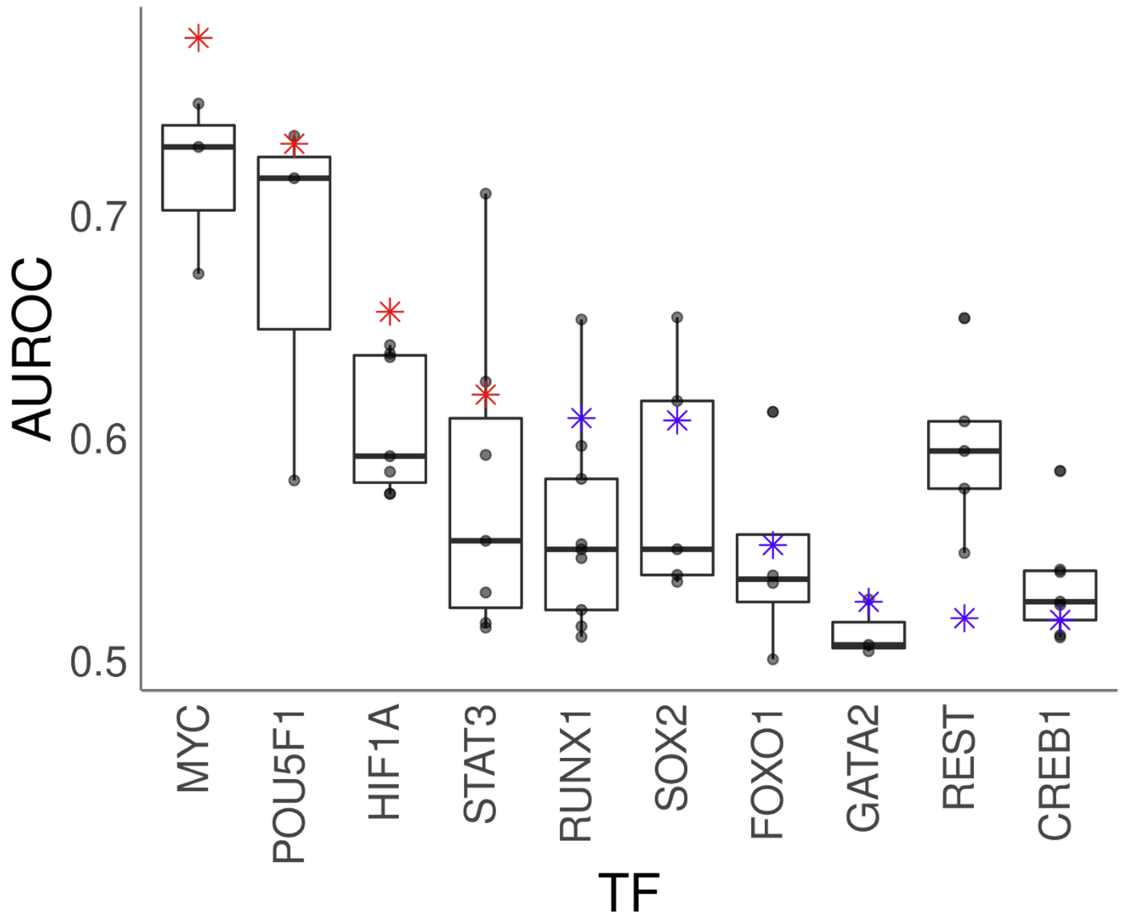

S21 Fig. Enrichment of curated targets in TF perturbation screens sourced from KnockTF. Each point is a high-throughput data set. Stars denote enrichment in the consensus ranking across all datasets for each TF. TFs are ordered by decreasing AUROCs in the consensus ranking. For eight of the 10 TFs, the consensus rankings are more enriched for the curated targets than the median across all datasets. Colors indicate statistical significance. For four TFs, the consensus rankings are significantly enriched for the curated targets.
