# Supplementary material for: Experiment level curation of transcriptional regulatory interactions in neurodevelopment
Source: PLoS Comput Biol. 2021 Oct 19;17(10):e1009484. doi: 10.1371/journal.pcbi.1009484 (PMC8565786; doi:10.1371/journal.pcbi.1009484)
Supplement: S22 Fig — AUROC enrichment values were computed for each unique combination of DTRI resource and TF where there 10 or more targets using the consensus rankings (See Methods). The size of each data point corresponds to the number of TFs where the AUROC values were computed. The data points show average AUROCs, and the error bars show the 95th confidence intervals. A confidence interval could not be computed for ENdb as this resource only contains enough (>10) targets for a single TF. (PDF) [file pcbi.1009484.s022.pdf]

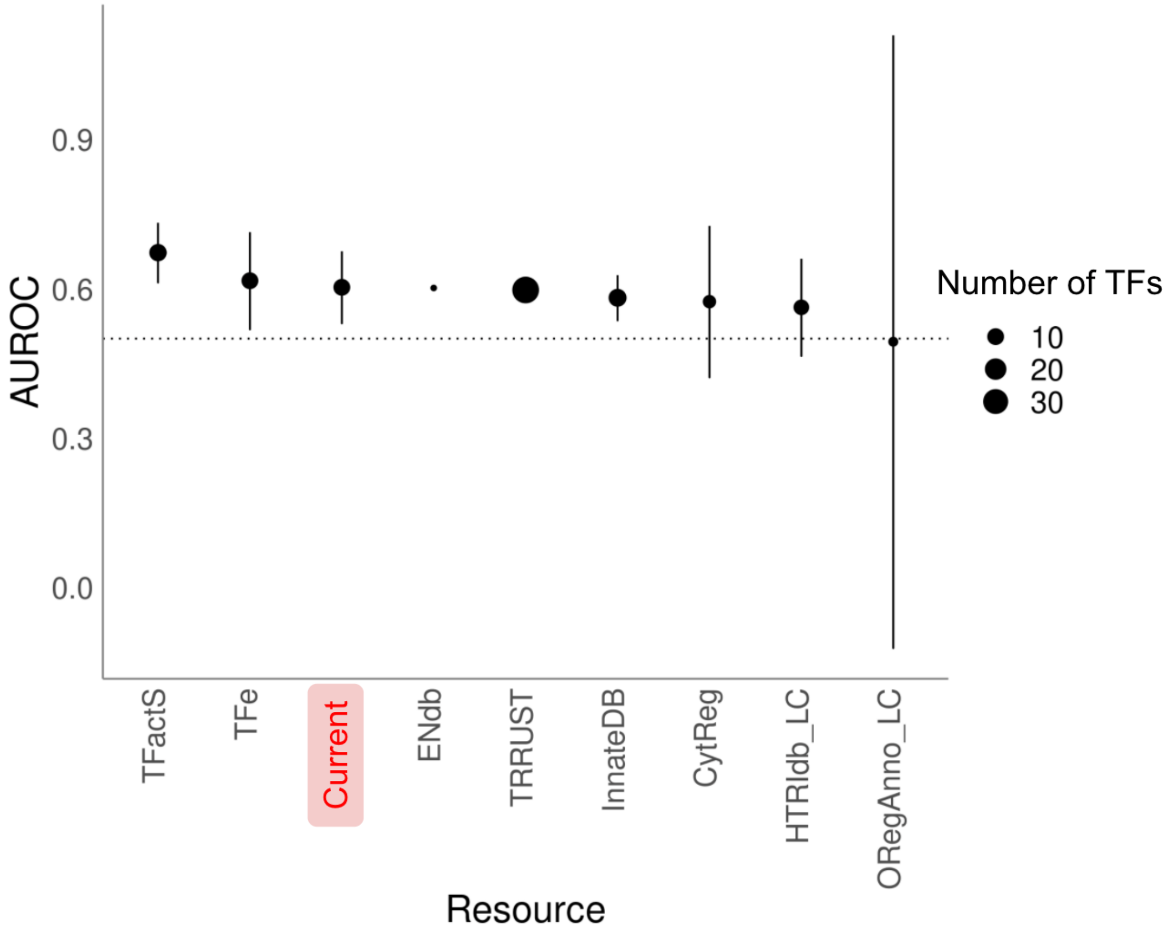

S22 Fig. Comparison of enrichment levels among DTRI resources. AUROC enrichment values were computed for each unique combination of DTRI resource and TF where there 10 or more targets using the consensus rankings (See Methods). The size of each data point corresponds to the number of TFs where the AUROC values were computed. The data points show average AUROCs, and the error bars show the 95th confidence intervals. A confidence interval could not be computed for ENdb as this resource only contains enough (>10) targets for a single TF.
