# Supplementary material for: Experiment level curation of transcriptional regulatory interactions in neurodevelopment
Source: PLoS Comput Biol. 2021 Oct 19;17(10):e1009484. doi: 10.1371/journal.pcbi.1009484 (PMC8565786; doi:10.1371/journal.pcbi.1009484)
Supplement: S1 Appendix — (PDF) [file pcbi.1009484.s036.pdf]

S1 Appendix. Curation Protocol

This document contains the protocol for reproducing the curation work described in the manuscript entitled “Experiment level curation of transcriptional regulatory interactions in neurodevelopment” by Chu et al. Part 1 describes the steps to assemble the corpus of candidate papers, by exploiting other resources of direct transcriptional regulatory interactions (DTRIs) and performing PubMed searches. The result of this is a large spreadsheet containing records of candidate papers that can be readily examined by curators. Part 2 includes descriptions of the annotations and the associated instructions for manually extracting these annotations from the research article in question.

Part 1: Candidate Papers Corpus Assembly

Sourcing candidate papers from external resources essentially entails generating a list of transcription factors (TFs) and PubMed ID mappings. The idea is to triage candidate papers that are likely to contain experimental evidence of DTRIs involving the annotated TF. To do this, first obtain the records of DTRIs from the target database in question (For example: TRRUST: (1,2), TFactS: (3), TFe: (4), etc). In most cases, each DTRI record would contain identifiers for the TF and target genes, usually as gene symbols, as well as a corresponding PubMed ID identifying the source article. Some resources will contain records that are outside the scope of this curation effort, which should be filtered out. For example, both OReganno (5–7) and HTRIdb (8) explicitly contain results from high-throughput studies. InnateDB (9,10) also contains protein-protein interactions and long non-coding RNA to target gene interactions that do not fit our definition of DTRIs. Next, to avoid ambiguity, all genes in this curation are to be indexed by Entrez IDs. To map the gene symbols or Ensembl IDs to Entrez IDs, use the gene annotations from NCBI for either human (https://www.ncbi.nlm.nih.gov/gene/?term=human%5Borganism%5D) or mouse (https://www.ncbi.nlm.nih.gov/gene/?term=mouse%5Borganism%5D). First, use the official HGNC or MGI gene symbols to map the Entrez IDs, and then by gene aliases. Drop the records with symbols that could not be mapped unambiguously. Finally, retain only unique TF to PubMed mappings. Repeat these steps for the different resources and then concatenate the results. Finally, add an additional column to keep track of the Entrez ID of the human ortholog to facilitate analysis. The resulting data table of candidate papers should have 5 columns, as shown in Table 1.

Table 1. Column descriptions of the table of candidate papers

Column	Details
TF_Symbol	**Example**: *PAX6*<br>**Description**: Symbol of the TF gene.
TF_Entrez_ID	**Example**: *5080*<br>**Description**: Entrez ID of the TF gene.

TF_Entrez_ID_Human	**Example**: *5080*<br>**Description**: Entrez ID of the TF's human ortholog. Note that this would be identical to TF_Entrez_ID if the human version was recorded in the source database.
PubMed_ID	**Example**: *12783797*<br>**Description**: PubMed ID identifier of the source article that may contain a DTRI report for the TF identified by TF_Entrez_ID, representing a candidate paper to be examined.
Database	**Example**: *TRRUST*<br>**Description**: The name of the source database from which this record was obtained.
Status	**Values**: *Curated, Examined, or Unexamined*<br>**Description**: Use this column to keep track of the status of curation of this candidate paper. Papers containing experimental evidence that have been curated should be marked '*Curated*'. Papers examined but not curated should be marked '*Examined*'. Papers not examined yet should be marked '*Unexamined*'.

Next, use the Gene Ontology (GO) and SFARI annotations to prioritize TFs relevant to neurodevelopment for curation. To produce the list of TFs, take the union set of all the TFs included in the external resources and the set of sequence specific TFs identified by Lambert et al. (11) (http://humantfs.ccbr.utoronto.ca/). Note that many external DTRI resources include co-TFs that do not have DNA binding domains. This is consistent with our definition of direct DTRIs where the TFs may associate with cis-regulatory elements (cREs) by interacting with other TFs or complexes. Once this set of TFs is assembled, obtain the set of human and mouse genes annotated with the neurodevelopment GO term (GO:0007417) from AmiGO (12,13) (http://amigo.geneontology.org/amigo). Similarly, obtain the set of genes annotated with Autism Spectrum Disorder (ASD) and Intellectual Disability (ID) associations in the SFARI database (14) (https://gene.sfari.org/). Join these tables to generate a list of TFs annotated with 7 columns, as shown in Table 2. These annotations can then be used to inform curation priority.

Table 2. Column descriptions of the TFs table

Column	Details
TF_Symbol	**Example**: *PAX6*<br>**Description**: Symbol of the TF gene.
TF_Entrez_ID	**Example**: *5080*

	**Description**: Entrez ID of the TF gene.
Go_Neurodev	**Value**: *TRUE or FALSE*<br>**Description**: A boolean indicating whether this TF was annotated with the central nervous system development GO term (GO:0007417), as indicated by the annotations from AmiGO.
SFARI_Score	**Value**: *1, 2, 3, or NA*<br>**Description**: A score indicating the confidence of this TF's association with ASD. Refer to the SFARI website (https://gene.sfari.org/about-gene-scoring/) for more details. If this TF was not annotated in SFARI, enter NA.
SFARI_Syndromic	**Value**: *TRUE or FALSE*<br>**Description**: A boolean indicating whether this TF is associated with a syndromic form of ASD.
Neurodev_TF	**Value**: *TRUE or FALSE*<br>**Description**: A boolean indicating whether this TF is associated with central nervous system development based on GO and SFARI.
N_Candidate_Paper	**Example**: *38*<br>**Description**: The number of candidate papers assigned to this TF. This number would be derived from the entire corpus of candidate papers including those sourced from external resources as well as the expansion from searching PubMed.

To expand the set of candidate papers, perform an independent PubMed search for each TF. Specifically, attach the following search string: "(((regulatory sequences, nucleic acid[MeSH Terms]) OR (transcription, genetic[MeSH Terms]) OR (intracellular signaling peptides and proteins[MeSH Terms]) OR (gene expression regulation[MeSH Terms])) AND ((Chromatin Immunoprecipitation[MeSH Terms]) OR (Electrophoretic Mobility Shift Assay[MeSH Terms])))" with the symbol of each TF gene, and use the E-Utilities API (15) (https://www.ncbi.nlm.nih.gov/books/NBK25501/) to perform the series of PubMed queries. Once ready, concatenate the results to construct a spreadsheet with the columns detailed in Table 1, making it compatible with the table of candidate papers retrieved from other DTRI resources. As such, the two sets would then be combined to form a master corpus of candidate papers. Once all the PubMed IDs are available, consider making additional queries to obtain and attach the paper titles and/or abstracts directly in the spreadsheet to facilitate curation.

Part 2: Manual Curation Guide

The remainder of this document describes the steps to extract and record experimental evidence of DTRIs from individual research articles. Here, we define a DTRI to be a transcriptional regulatory interaction where the TF protein first binds (either directly or indirectly through a co-binding partner) to a cRE that lies near the target gene, and consequently influences the level of its transcription. This is in contrast with “indirect regulation” where multiple DTRIs act in series (for example: Pax6 > Neurog2 > Sox2, where the relationships between Pax6 and Sox2 would be considered “indirect”). Importantly, we will curate only low-throughput reports of DTRIs at the individual experiment level. Each experiment will be represented by an independent record in the resulting database, annotated with attributes that are specific to that experiment. Further, consistent with our focus on mammalian neurodevelopment, this curation will only record experiments performed in humans or mice.

The first step of curation is to identify the low-throughput experiments that lend support to DTRIs. Specifically, we are interested in three types of experiments: 1. TF perturbation, 2. TF-DNA binding, and 3. TF-reporter assays. In order for a TF to directly regulate a target gene, the TF protein must bind to a regulatory site near the target gene’s TSS. However, while TF-DNA association is necessary, it is insufficient to cause regulation. Therefore, to establish a DTRI, it must also be demonstrated that the binding can activate the corresponding cRE and influence the target gene’s level of transcription. As such, the three types of experiments taken together can provide stronger evidence for direct transcriptional regulation.

In a TF perturbation experiment, an induced change in the expression of the regulator gene is shown to lead to a change in the expression of the target gene. This usually involves perturbing the TF expression followed by measuring target gene expression. For example, Sun et al. looked at the difference in Neurog2 expression between Pax6-null and wild type mouse forebrains using RT-PCR (16). Note that different perturbation and measurement strategies may be used. TF-DNA binding experiments measure the interaction between the TF protein and the TFBS in question. This could be shown using a ChIP experiment or an electromobility shift assay (EMSA). For example, in the same study, Sun et al. used a ChIP assay to examine the binding of the Pax6 protein near the target gene Neurog2. Finally, TF-reporter experiments provide experimental evidence that the TF regulator activates the TFBS. This is usually performed by measuring the activity of a reporter assay that is driven by the corresponding TFBS in the presence or absence of the TF protein. For example, Scardigli et al. co-electroporated a reporter construct containing the E1 enhancer with a Pax6 expression vector into chick embryos (17). They found that ectopic expression of Pax6 drove the expression of the reporter gene, in comparison to the control.

The curation process largely involves going through the full text of candidate papers for a given TF, and possibly supplementary materials, reviewing each one to identify the set of experiments reported, and creating entries in the curation sheets to document the

experimental details. Before delving deeply into a paper, it is often useful to triage papers by reading only the title and the abstract. By doing this, it may be apparent whether the paper contains support for one or more DTRIs using low-throughput experimental evidence. For example, the title or the abstract may indicate that only high-throughput evidence is reported. In that case, it would be safe to mark the paper as “examined” and carry on. Once a paper is determined to be likely to contain low-throughput experiment evidence, get the full text and identify the experiments performed. The experiments are often laid out in the results section though it may be necessary to also look at the methods and/or the supplementary materials to extract the relevant details. In favour of efficiency and thoroughness, curate all experiments reported by the paper under inspection, even if it involves a different TF than expected.

In total, there are 4 curation spreadsheets. The rows correspond to individual experiments and the columns contain the various attributes to be recorded. The first spreadsheet is the master curation sheet containing basic information for all experiments, regardless of the type of experiment (Table 3). Each experiment will have an additional entry in one of the other 3 curation sheets based on the type of assay. A unique identifier should be assigned to each experiment at the time of curation so that entries could be mapped across tables. Controlled vocabularies are used for all recorded attributes to simplify the curation process. Refer to the curation tables below for instructions on how to record each attribute.

Table 3. Column descriptions of the master curation sheet

Column	Details
**Experiment_ID**	**Example**: *Exp_001*<br>**Required**: Yes<br>**Description**: A unique identifier for this experiment. This is used to map the experiment to other spreadsheets containing additional details. Additional spreadsheets are necessary as the details to be annotated are different for each experiment type.
**TF_Entrez_ID**	**Example**: *5080*<br>**Required**: Yes<br>**Description**: The NCBI Entrez ID for the TF regulator gene. To avoid ambiguity, do not use gene symbols. In case the Entrez ID could not be identified in the original article, use the NCBI website (https://www.ncbi.nlm.nih.gov/gene) for conversion. Note that the Entrez ID implicitly encodes the species information. For example, the mouse Pax6 gene is 18508 whereas the human PAX6 gene is 5080. Make sure that the Entrez ID for the appropriate species is recorded. Except in TF perturbation or ChIP assays, the species of the TF protein is not necessarily the same as the species of the

	target TFBS.
**Target_Entrez_ID**	**Example**: *6657*<br>**Required**: Yes<br>**Description**: The NCBI Entrez ID for the target gene. Refer to the instructions for TF_Entrez_ID.
**PubMed_ID**	**Example**: *12783797*<br>**Required**: Yes<br>**Description**: The PubMed ID of the research article reporting this experiment.
**Experiment_Type**	**Value**: *TF Perturbation, TF-DNA Binding, or TF-Reporter*<br>**Required**: Yes<br>**Description**: One of the three types of experiments being curated. This annotation will determine the types of details to be recorded in one of the other spreadsheets, described in Tables 4-5.
**Context_Type**	**Value**: *Primary Tissue, Primary Cells, Cell Line, or In Vitro*<br>**Required**: Yes<br>**Description**: A broad classification of the cellular context tested. This annotation is applicable across all three types of experiments. Note that TF Perturbation, TF-Reporter, and ChIP assays cannot be in-vitro as they must be carried out in live cells. In contrast, EMSAs are performed in vitro.
**Cell_Type**	**Example**: *UBERON:0001017*<br>**Required**: Only for primary tissue, primary cells, or cell lines<br>**Description**: An ontology term that best corresponds to the tissue or cell type used. If the context type is in vitro, enter *'Not Applicable'*. For primary tissue, use a term from the Uberon ontology (18). Consider using the EBI website (https://www.ebi.ac.uk/ols/ontologies/uberon) to search for the most appropriate ontology term. Always strive to retain the highest possible resolution. For example, if an experiment was performed using the forebrain (UBERON:0001890), do not annotate it simply as the central nervous system (UBERON:0001017). For primary cells, use terms from the CL ontology (19) (https://www.ebi.ac.uk/ols/ontologies/cl). Finally, for cell lines, use the CLO ontology (20) (https://www.ebi.ac.uk/ols/ontologies/clo). Where the appropriate term could not be found in the

	aforementioned ontologies, consider trying alternatives such as the BTO (21) or EFO (22) ontologies. As a last resort, enter the free text directly from the original article.
**Age**	**Example**: *E14*<br>**Required**: Only for primary tissue or primary cells<br>**Description**: Age of the model organism. Use '*E*' or '*P*' to indicate embryonic or postnatal, followed by a number to indicate the number of days. For example, embryonic day 14 is encoded as E14 whereas postnatal day 5 is encoded as P5. If this information could not be found, enter *'Unknown'*. Enter *'Not Applicable'* for cell lines.
**Negative_Case**	**Value**: *TRUE or FALSE*<br>**Required**: Yes<br>**Description**: Did this experiment yield a negative finding? Enter TRUE if this is the case, otherwise FALSE.

Table 4. Column descriptions of the TF perturbation details curation sheet

Column	Details
**Experiment_ID**	**Example**: *Exp_001*<br>**Required**: Yes<br>**Description**: A unique identifier for this experiment. This is used to map experiments back to the master curation table.
**Mode**	**Value**: *Activation or Repression*<br>**Required**: Yes<br>**Description**: The mode or direction of regulation. If the direction of change in the resulting target gene expression is the same as the direction of change in the TF gene expression, then enter *'Activation'*, for example, if the TF is knocked out, and the target gene expression diminishes. If the change in target gene expression is opposite to that of the TF, then enter *'Repression'*.
**Effect**	**Value**: *Homozygous Knockout, Heterozygous Knockout, Knock Down, or Overexpression*<br>**Required**: Yes<br>**Description**: The effect of experimental manipulation. Knockouts refer to DNA mutations. This may be achieved by using naturally occurring mutations, as in the case of the Pax6-SEY mutants in the study by Scardigli et al. (17), or induced

	experimentally by using Cre-Lox strategies, CRISPR, etc. Missense mutations may also be classified as genetic knockouts if the resulting effect is loss of function, as indicated by the original article. Knockouts could be further classified as homozygous or heterozygous. Enter this information if available. Knockdown refers to strategies that down regulate the TF protein without inducing genetic modifications. For example, Easwaran et al. knocked down CTNNB1 expression by using RNA interference (23). Finally, overexpressions are when the TF protein is upregulated, for example, by delivering plasmids for ectopic transcription.
**Type**	**Value**: *Constitutive or Induced*<br>**Required**: Yes<br>**Description**: Constitutive perturbation refers to mutations that are present throughout the course of development and are present in all cells, as opposed to induced perturbations using Cre-Lox or RNA interference that may be triggered closer to the time of assay and may have tissue or cell type specificity.

Table 5. Column descriptions of the TF-DNA binding details curation sheet

Column	Details
**Experiment_ID**	**Example**: *Exp_001*<br>**Required**: Yes<br>**Description**: A unique identifier for this experiment. This is used to map experiments back to the master curation table.
**TFBS**	**Example**: *-450*<br>**Required**: No, enter *'Unknown'* if this information could not be found.<br>**Description**: Distance of the TFBS from the target gene's TSS. Usually the TFBS' distance is reported as an interval with two genomic coordinates, relative to the target gene's TSS. Enter the end that is closer to the target TSS. A minus sign (-) indicates upstream and a plus sign (+) indicates downstream. For example, in the study by Scardigli et al., the tested E1 element is at coordinates [-9368, -7610] in the Neurog2 locus (17). In this case, enter *-7610*. Note that this piece of information may not be provided at the base pair level. If so, enter rounded values where available. If

	the paper simply indicates the TFBS is in the target gene's promoter, enter '*Promoter*'.
**Method**	**Value**: *ChIP or EMSA*<br>**Required**: Yes<br>**Description**: The type of assay used to investigate binding. This is usually ChIP or EMSA.
**TF_Source_Type**	**Value**: *Primary Tissue, Primary Cells, Cell Line, or In-Vitro*<br>**Required**: Only for EMSA experiments<br>**Description**: The cellular context from which the TF molecules were obtained for this experiment.

Table 6. Column descriptions of the TF-reporter details curation sheet

Column	Details
**Experiment_ID**	**Example**: *Exp_001*<br>**Required**: Yes<br>**Description**: A unique identifier for this experiment. This is used to map experiments back to the master curation table.
**Mode**	Refer to Table 4 for instructions.
**TFBS**	Refer to Table 5 for instructions.
**Mutated**	**Value**: *True or False*<br>**Required**: Yes<br>**Description**: A boolean to indicate whether this experiment also characterized the effect of mutations in the TFBS sequence in their reporter assay?
**Binding_Verified**	**Value**: *Putative or EMSA*<br>**Required**: Only for cases where Mutated is '*TRUE*'<br>**Description**: Use this annotation to indicate whether the experiment demonstrated that the mutation results in a difference in the TF-DNA binding affinity using additional EMSA experiments. If the disruption was not experimentally verified, enter *'Putative'*.

Citations

1. Han H, Cho J-W, Lee S, Yun A, Kim H, Bae D, et al. TRRUST v2: an expanded reference database of human and mouse transcriptional regulatory interactions. Nucleic Acids Res. 2018 Jan 4;46(D1):D380–6.

2. Han H, Shim H, Shin D, Shim JE, Ko Y, Shin J, et al. TRRUST: a reference database of human transcriptional regulatory interactions. Sci Rep. 2015 Jun 12;5(1):11432.

3. Essaghir A, Toffalini F, Knoops L, Kallin A, van Helden J, Demoulin J-B. Transcription factor regulation can be accurately predicted from the presence of target gene signatures in microarray gene expression data. Nucleic Acids Res. 2010 Jun;38(11):e120–e120.

4. Yusuf D, Butland SL, Swanson MI, Bolotin E, Ticoll A, Cheung WA, et al. The Transcription Factor Encyclopedia. Genome Biol. 2012 Mar 29;13(3):R24.

5. Lesurf R, Cotto KC, Wang G, Griffith M, Kasaian K, Jones SJM, et al. ORegAnno 3.0: a community-driven resource for curated regulatory annotation. Nucleic Acids Res. 2016 Jan 4;44(D1):D126-132.

6. Griffith OL, Montgomery SB, Bernier B, Chu B, Kasaian K, Aerts S, et al. ORegAnno: an open-access community-driven resource for regulatory annotation. Nucleic Acids Res. 2008 Jan 1;36(suppl_1):D107–13.

7. Montgomery SB, Griffith OL, Sleumer MC, Bergman CM, Bilenky M, Pleasance ED, et al. ORegAnno: an open access database and curation system for literature-derived promoters, transcription factor binding sites and regulatory variation. Bioinformatics. 2006 Mar 1;22(5):637–40.

8. Bovolenta LA, Acencio ML, Lemke N. HTRIdb: an open-access database for experimentally verified human transcriptional regulation interactions. BMC Genomics. 2012;13(1):405.

9. Breuer K, Foroushani AK, Laird MR, Chen C, Sribnaia A, Lo R, et al. InnateDB: systems biology of innate immunity and beyond--recent updates and continuing curation. Nucleic Acids Res. 2013 Jan;41(Database issue):D1228-1233.

10. Lynn DJ, Winsor GL, Chan C, Richard N, Laird MR, Barsky A, et al. InnateDB: facilitating systems-level analyses of the mammalian innate immune response. Mol Syst Biol [Internet]. 2008 Sep 2 [cited 2013 Jun 5];4(1). Available from: http://www.nature.com/msb/journal/v4/n1/full/msb200855.html

11. Lambert SA, Jolma A, Campitelli LF, Das PK, Yin Y, Albu M, et al. The Human Transcription Factors. Cell. 2018 Feb 8;172(4):650–65.

12. Carbon S, Ireland A, Mungall CJ, Shu S, Marshall B, Lewis S. AmiGO: online access to ontology and annotation data. Bioinformatics. 2009 Jan 15;25(2):288–9.

13. Gene Ontology Consortium. The Gene Ontology (GO) database and informatics resource. Nucleic Acids Res. 2004 Jan 1;32(suppl_1):D258–61.

14. Abrahams BS, Arking DE, Campbell DB, Mefford HC, Morrow EM, Weiss LA, et al. SFARI Gene 2.0: a community-driven knowledgebase for the autism spectrum disorders (ASDs). Mol Autism. 2013 Oct 3;4(1):36.

15. NCBI Resource Coordinators. Database Resources of the National Center for Biotechnology Information. Nucleic Acids Res. 2017 Jan 4;45(D1):D12–7.

16. Sun J, Rockowitz S, Xie Q, Ashery-Padan R, Zheng D, Cvekl A. Identification of in vivo DNA-binding mechanisms of Pax6 and reconstruction of Pax6-dependent gene regulatory networks during forebrain and lens development. Nucleic Acids Res. 2015 Aug 18;43(14):6827–46.

17. Scardigli R, Bäumer N, Gruss P, Guillemot F, Roux IL. Direct and concentration-dependent regulation of the proneural gene Neurogenin2 by Pax6. Development. 2003 Jul 15;130(14):3269–81.

18. Mungall CJ, Torniai C, Gkoutos GV, Lewis SE, Haendel MA. Uberon, an integrative multi-species anatomy ontology. Genome Biol. 2012 Jan 31;13(1):R5.

19. Diehl AD, Meehan TF, Bradford YM, Brush MH, Dahdul WM, Dougall DS, et al. The Cell Ontology 2016: enhanced content, modularization, and ontology interoperability. J Biomed Semant. 2016 Jul 4;7(1):44.

20. Sarntivijai S, Lin Y, Xiang Z, Meehan TF, Diehl AD, Vempati UD, et al. CLO: The cell line ontology. J Biomed Semant. 2014 Aug 13;5(1):37.

21. Gremse M, Chang A, Schomburg I, Grote A, Scheer M, Ebeling C, et al. The BRENDA Tissue Ontology (BTO): the first all-integrating ontology of all organisms for enzyme sources. Nucleic Acids Res. 2011 Jan 1;39(suppl_1):D507–13.

22. Malone J, Holloway E, Adamusiak T, Kapushesky M, Zheng J, Kolesnikov N, et al. Modeling sample variables with an Experimental Factor Ontology. Bioinformatics. 2010 Apr 15;26(8):1112–8.

23. Easwaran V, Lee SH, Inge L, Guo L, Goldbeck C, Garrett E, et al. beta-Catenin regulates vascular endothelial growth factor expression in colon cancer. Cancer Res. 2003 Jun 15;63(12):3145–53.
