# Supplementary material for: Experiment level curation of transcriptional regulatory interactions in neurodevelopment
Source: PLoS Comput Biol. 2021 Oct 19;17(10):e1009484. doi: 10.1371/journal.pcbi.1009484 (PMC8565786; doi:10.1371/journal.pcbi.1009484)
Supplement: Attachment — Submitted filename: response_letter.docx [file pcbi.1009484.s037.docx]

To the Editor,

Please consider the revised manuscript entitled “Experiment level curation of transcriptional regulatory interactions in neurodevelopment” by Chu et al. for publication as a research article in PLoS Computational Biology. We thank the editors and reviewers for a thorough review of our work and the valuable feedback.

We are excited to receive affirmation from both reviewers that we have produced a “valuable resource” (Reviewers 1 & 2) with “commendable richness in experimental and biological context detail” (Reviewer 2). A primary concern raised by both reviewers stems from the unfortunate impression we gave in the initial submission that we were making a broad claim of superiority over other resources, without sufficient validation. We regret that our wording in a few places encouraged this unintended interpretation. In response to the reviewer’s suggestion, in addition to tuning the wording we have added multiple validations and comparisons that help put our work in context. We feel these changes substantially strengthen the manuscript to more accurately and specifically emphasize the aspects in which our work has unique and complementary value.

Dr. Lægreid (Reviewer 2) has additionally provided several helpful suggestions regarding definitions of terminology, clarity of writing, data formats, as well as documentation of the curation protocol. We have addressed each of these concerns in the revised manuscript.

During our revisions of the manuscript, we discovered two minor errors. We have corrected these in the revision, though neither affects our conclusions. The first error concerns the difference in the AUROC enrichment levels between the targets annotated with multiple types of experiments versus those with a single type (S17 Fig). In the initial submission, the manuscript stated that the difference is statistically significant (Manuscript Line 440). While the magnitude of the difference is large for the hit list analysis (2.6-fold), the difference in AUROC enrichment did not pass the threshold for statistical significance according to our resampling test. We have now corrected this in the manuscript as well as in the legend of S17 Fig. A second error involves curation of TFBS coordinates. A very small fraction (less than 10) of the records was annotated using absolute genomic coordinates and mistakenly classified as “distal TFBSs” based on the magnitude of the number. We have now converted these numbers into the intended units (relative to target genes’ TSSs) and updated the data in S3 Data and figures S11 Fig and S12 Fig. Again, neither of these errors had an impact on the conclusions of this paper.

Our point-by-point response follows.

Reviewer #1:

*In this manuscript the authors describe the construction of a manually curated collection of TF-gene regulatory interactions, involving 251 TFs associated with nervous system development. Although being restricted to a small set of TFs of interest, this work provides a valuable resource for the community, containing important information such as type of experimental evidences of the TF-gene interactions and cellular context, adding to the novelty of the described database compared to the published resources. The analyses are generally well performed, the curation protocol is well described and all data and source code is made available. Overall, this study merits publication in PLOS Computational Biology. I have however some comments that I believe need to be addressed.*

We thank the reviewer for the positive feedback. We recognize that this resource is currently limited to a small set of TFs. One of the goals of this study was to develop and pilot the curation protocol (S1 Appendix) as a proof-of-principle for a more scaled-up undertaking. We have, in fact, recently been funded by the NIH to expand this curation effort substantially. We are excited to be able to use the feedback from the reviewers and the community to guide our future work.

*1) Authors validate their database using one example of a Pax6 perturbation study. To strengthen their approach, they should consider providing 1-2 more examples of other TFs for which there is available perturbation data.*

Our inclusion of the comparison with a single high-throughput Pax6 perturbation screen was intended to be a demonstration of a potential use case of our resource, with an illustration of the value of detailed curation rather than as formal validation of the TF-gene relationships reported in the literature. Ultimately, any resource based on literature curation is only as good as that literature, and we were not attempting to validate that literature by comparing it to a high-throughput experiment. Instead, we were modeling the use case of trying to validate a high-throughput experiment by appealing to the low-throughput literature. By capturing the experimental details, we equip the users with more granular information than other resources, simplifying independent assessments of reliability of the underlying data. Our demonstration shows how knowing the experimental context allows making a more specific evaluation of the Pax6 perturbation by isolating gene targets identified in the relevant tissue (embryonic brain). If the results of this demonstration had been negative, we would have been driven to question the quality of either the Pax6 perturbation study data or our curation (or both), so there is a germ of truth to the idea that this example “validates” our curation and the underlying literature to an extent, but that was not our main intent. In response to the suggestion, we have modified our title, and writing (Manuscript Lines 28, 31, 52, 59-60, 113-119, 419-478) to clarify the goals of the analysis in Fig 5 and S17 Fig. To make sure we fully address the reviewer’s suggestion, we have also replicated the results in an additional PAX6/Pax6 perturbation dataset of a similar biological context (S19 Fig, S20 Fig). Note that we could not repeat the same analysis using other TFs because of the sparsity in tissue specific target coverage among TFs other than PAX6/Pax6. In addition, we also used KnockTF (PMID: 31598675) to perform a series of comparisons with 10 other TFs, aggregating across 52 datasets (S21 Fig).

*2) To compare the performance of their approach with the available databases, the authors should provide Pax6 targets enrichment results using 1-2 other published resources they mention, e.g. TRRUST or CytReg.*

We agree that it would be interesting to compare the various curation resources. However, this is difficult due to the generally small scale of DTRI resources, including ours. TRRUST is the only other resource with more than 10 targets curated for PAX6. The other databases range from 0 (HTRIdb, InnateDB, ORegAnno) to 9 (TFe). We have now included TRRUST in the PAX6 analyses (S17 Fig). Here, we observed higher level of enrichment among CNS targets than the targets recorded in TRRUST, again demonstrating the utility of our tissue annotations. Our analyses of KnockTF perturbation datasets enabled comparison with additional curation resources for a small number of TFs (S22 Fig). Based on this limited set of comparisons, we conclude that our resource consists of DTRIs with detectable correspondence with high-throughput data and that the level of correspondence is comparable with other similar resources.

*3) Minor comment: Figures 2A and 4D – colours of the Venn diagrams are too dim, making the diagrams not visible.*

We have adjusted the colors of these figures to improve visibility.

Reviewer #2:

*The availability of high quality curated regulatory information annotated with evidence from low-throughput experiments and information regarding mode of regulation and biological context is of high importance for all aspects of life sciences that employ computational approaches involving comprehensive querying and modelling of digital standardised data, information and knowledge with the aim to seek improved understanding of complex biological systems to optimally support research and innovation for societal sectors, including health care, food, biotech industry and environment. Biocuration, as accomplished in the current work - the extraction of knowledge from unstructured biological data into a structured, computable form – is scientific work of high importance and impact. The large set of information regarding transcriptional regulatory interactions involving transcription factors with roles in neurodevelopment generated by the authors represents a highly valuable resource, in particular for the field of neurobiology. Specifically, the curated compendium of information is of commendable richness in experimental and biological context detail, all of which is systematically mapped to acknowledged ontologies and controlled vocabularies. Moreover, the paper includes analyses of the curated information allowing for prudent and informative discussion of research bias embodied in the scientific literature and challenges related to lack of completeness of information on transcriptional regulatory interactions in existing databases.*

We thank the reviewer for the positive feedback.

*The authors hypothesize that information regarding direct transcriptional regulatory interactions (DTRIs) evidenced by low-throughput experiments as documented by curation of scientific literature, can produce a reliable set of DTRIs that is highly interpretable and more suitable for the evaluation of high-throughput predictions than other, similar resources. The group conducted a curation effort to generate a set of 1499 DTRIs focussing 251 transcription factors (TFs) considered to be involved in neurodevelopment, and for which evidence from low-throughput experiments could be found in the scientific literature. The hypothesis was then tested by comparing the DTRIs in the curated set with the content in existing resources and with transcriptional regulatory interactions obseved in a previousy published high-throughput Pax6 perturbation screen. Based on finding an enrichment of curated, experimentally evidenced Pax6 DTRIs among the transcriptional interactions detected in the high-throughput screen, the authors claim that the literature curated set of DTRIs represents a reliable knowledge resource with high sensitivity for DTRIs predicted from HTP screens and more suitable for the evaluation of high-throughput predictions than other, similar resources. Furthermore, the authors claim that their curated set af DTRIs is more interpretable than other similar resources.*

*The originality of the claims is modest, since it is widely accepted that information and knowledge manually curated from full text papers where it is normally supported by low-throughput experimental evidence has high value for the current life sciences. This has been widely discussed and documented, amongst others through the activites of the Gene Ontology Consortium and IMEX Consortium.*

*Specifically, the value of such information and knowledge in the field of transcriptional regulatory interactions, has been convincingly demonstrated by Garcia-Alonso L, et al, 2019 (PMID: 31340985). An in depth manual curation effort for transcriptional regulatory interactions within the subdomain of liver-enriched TFs by e.g. Thomas P, et al, 2015 (PMID: 25433699) similarly demonstrated such value.*

*The manuscript’s support for the claims that the curated set of DTRIs is highly reliable and is more interpretable and more suitable than other, similar resources for the evaluation of predictions from high-throughput experiments is on the weak side. This is related to the circumstance that set of DTRIs has only been tested against one HTP screen probing TF Pax6 transcriptional regulations and that the attribute ‘interpretability’ has not been rigorously defined and tested.*

As mentioned in the preamble of this letter, we regret that some of the writing in our initial submission had given the impression of claiming superiority over other DTRI resources particularly for evaluating high-throughput predictions. As detailed in our response to Pts.1 & 2 by Reviewer 1, we did not intend for the comparison with the single Pax6 perturbation screen to be understood as a validation of our resource but as demonstration of a potential use case. Importantly, we propose that the main value provided by our curation is the level of granularity in the experimental and contextual details captured about each TF-target relationship. As part of this revision, we have amended the writing for clarity. Further, to ensure that we fully address the reviewer’s concerns, we have also added additional high-throughput comparisons to help put our work in context.

*1) The curated set of DTRIs should be tested against additonal HTP screens representing a wider repertoir of TFs, and ideally including more than one type of HTP analysis (for instance ChIP in addition to transcriptomics) to derive more robust support for the claim pertaining to suitability for evaluation of high-throughput predictions.*

Please see our response to Pts. 1 & 2 by Reviewer 1 for clarification on our claims and the additional comparisons with high-throughput TF perturbation studies. While we agree that further comparisons with additional types of high-throughput data (ChIP-seq, DNase-seq and others) is an interesting question, a comprehensive assessment is beyond the scope of this study. Based on the additional comparisons we performed, we conclude that our resource consists of DTRIs with detectable correspondence with high-throughput TF perturbation data (S21 Fig) and that the level of correspondence is comparable with other similar resources (S22 Fig).

*2) To support the claim pertaining to higher interpretability, it would be necessary for the authors to define what they mean by interpretability. This should be followed by testing against other, similar resources.*

We regret that our use of the word “interpretability” was unclear. We were referring to the systematic curation of details at the individual experiment level, which means TF-gene relations can be interpreted in terms of context, experimental method, and more. To the best of our knowledge, no previous curation efforts have captured this level of granularity, forming the basis of our claim of higher interpretability, but we expressed this clumsily. For example, TRRUST used a semi-automated text mining approach to identify DTRIs from publication abstracts, purposely overlooking the underlying experiments. TFe relied on expert assessment and curation of DTRIs without recording other details. TFactS, OReganno, CytReg, InnateDB, HTRIdb, and ENdb aggregated DTRIs supported by single experiments, which are commonly the TF-DNA binding assay. Many of these resources also included DTRIs tested using high-throughput methods, either purposely or unintentionally. Finally, the only resources that recorded tissue context were ENdb and InnateDB, both of which documented this information at the DTRI level. In this revision, we have removed use of the term “interpretability” in favor of more specific terminology (Manuscript Lines 113-119, 490-491). We have also added more details to the third paragraph (Manuscript Lines 505-525) in the discussion section, which already contains elaborations on the differences in the annotated information among the different resources, which addresses the request by the reviewer to compare to other resources on this issue.

*3) Of central focus of the presented work is the curation of «direct transcriptional regulatory interactions (DTRIs)». However, the authors do not specify what they mean by «direct». According to established conventions, the regulators involved in «direct» transcription regulation are regarded to be the DNA -binding transcription factors (dbTFs) whereas the transcription co-regulators are considered to be more indirectly involved via protein interactions with dbTFs. GO-terms GO:0003700 and GO:0003712 can be consulted for authoritative definitions of the dstinct molecular functions of dbTFs and co-TFs. The TFs in the curated DTRI set comprises regulators of both categories. Examples of proteins in the set that are considered to be co-TFs and not dbTFs are CTNNB1 and SPOP. The reviewer is aware that term «transcription factor» is often used for both dbTFs and co-TFs, also in several of the existing databases used and cited in the current work. Nevertheless, it is of high importance for use and interpretation of the valuable resource generated in the present work that it is explicitly stated what is meant by «direct», and to enable users of the data to adequately deal with dbTFs and co-TFs in the gene regulatory networks enabled by the resource.*

We regret the confusion. In our study, we define a direct regulatory interaction to be between a TF and a gene it regulates by associating with a cRE for that gene, whether by binding to DNA itself or by interaction with one or more other molecules which are in turn binding to the cRE. In other words, the “directness” comes not necessarily from physical contact with the DNA but from the logic of the regulatory circuit. Under this definition, an “indirect” relation is one with one or more intermediate direct regulatory interactions acting in series, for example TF A activates TF B which in turn activates effector gene C - the relation between TF A and gene C is “indirect” in our meaning, while the relations between TF A and TF B, and between TF B and gene C, are two “direct” regulatory interactions. This use of the term “direct” is not unique to our work, as evidenced by the inclusion of co-TFs in other DTRI resources. Nonetheless, we appreciate the confusion and have now clarified this in the manuscript (Manuscript Lines 80-84).

*4) Related to the issue discussed in 3), the authors should clarify the relevance and confidence of a direct regulatory interaction investigated by ChIP- and reporter experiments, compared to experiments like EMSA performed with purified TFs. The reviewer regards the latter as a clearly stronger evidence for direct protein-gene interaction than the former, even though some reporter gene experimental set ups, e.g. those utilizing gene regulatory regions specifically mutated in the TFBS, can also provide strong indication for direct interaction. The reviewer disagrees with the authors that «…reliability of EMSAs might be improved by using the endogenous TF protein…» in nuclear extracts (manuscript line 232-). It is commonly thought that it is not possible to ascertain the nature of the protein (complex) represented in an EMSA band shift produced from nuclear extracts, meaning that it is not known which protein species are involved, and which specific protein(s) directly bind the DNA.*

As we have clarified above, our focus is on “direct’ in the sense of regulatory logic, rather than on “direct” DNA binding. We agree that EMSA is a good way to establish binding, though there are always caveats to studies performed in reconstituted systems *in vitro*. As the reviewer appreciates, binding is not guaranteed to result in regulation, so assays like reporters can have an important role. As our intent is capturing in vivo regulatory logic, one could argue that ChIP and reporter assays might carry more weight. However, as previously stated, we emphasize that by making the annotations available, we equip the users to derive their own assessments of confidence. We have reworded the relevant paragraphs in the results section in favour of neutrality on the issue of which, if any, forms of evidence are stronger, as well as for other collected attributes (Manuscript Lines 243-266). Further, we thank the reviewer for pointing out that EMSAs using nuclear extracts alone cannot unambiguously implicate the TF protein in question. We have removed the associated statements (Manuscript Line 260).

*5) The manuscript refers to the curation protocol developed for this work. However the reviewer was unable to find this protocol in the submitted material. The protocol should be made available in a form that would enable a better understanding of how the curated information was identified and mapped to the chosen ontologies and controlled vocabularies and to enable other curators to reproduce the developed methodology for similar curation efforts. It would be highly preferable that the protocol includes guidelines pertaining to which sections of the full paper (e.g. result section, figures, method sections) to be used for the different curation aspects and how the identified information should be used to generate the appropriate annotations.*

Thank you for the suggestion. We have included the complete protocol in S1 Appendix. Note that this is adapted from our internal Wiki, so it is not identical to the documentation used in the project, but accurately represents the processes we followed.

*6) The definition of proximal and distal elements applied in the current work is not in line with the most updated definition for proximal-distal TFBS provided by the highly authoritative Sequence Ontology (SO). For example, the SO defines a proximal TFBS (SO:0001668) to be located in the region from about -250 to -40 relative to +1 of RNA transcription start site. The authors should consider to apply the definitions laborated by SO, or at least comment on how they deviate from this definition.*

We realize that any threshold of calling a TFBS proximal vs. distal is largely arbitrary. However, we must clarify that the purpose of making this distinction is to document research biases among studies of TFBSs, rather than to distinguish between promoters and enhancers. The strict definition of “proximal TFBS” (SO:0001668) noted by the reviewer is in fact the definition of proximal promoters, not TFBSs in general. Further, note that ENCODE designates anything <2kb as “proximal cREs” (<https://screen.encodeproject.org/>; PMID: 32728249). We have now adopted the ENCODE standard of 2kb for calling proximal vs. distal TFBSs. As expected, this did not impact the pattern of research bias that we had identified. To reinforce this observation, we have now also included an additional supplementary figure (S13 Fig) showing the distribution of experiments across TFBS coordinates.

*7) The data of the paper are made available in large detail and depth in supplementary files. However, in order to maximally enable the re-use of this valuable information, the authors are strongly adviced to re-check how the presentation of the curated data complies with established standards for representing regulatory interactions with the aim to implement adjustments that could enhance interoperability with other resources. Such standards have been formulated and discussed by e.g. the Gene Ontology Consortium, 2021 (PMID: 33290552), Perfetto et al, 2019 (PMID: 30793173) and Touré et al, 2021 (PMID: 33547799). The PSICQUIC exchange format developed by the EBI is well suited for dissemination of regulatory interaction information and could be considered for use by the authors in order to optimally support re-use and comply with FAIR principles.*

We have provided a version of the data using the controlled vocabulary provided by PSI-MI (<https://www.ebi.ac.uk/ols/ontologies/mi>; S12 Data), in addition to our original formats. However, we stress that this format loses much of the information we capture in our curation in terms of experimental and contextual detail.
