# Supplementary material for: Experiment level curation of transcriptional regulatory interactions in neurodevelopment
Source: PLoS Comput Biol. 2021 Oct 19;17(10):e1009484. doi: 10.1371/journal.pcbi.1009484 (PMC8565786; doi:10.1371/journal.pcbi.1009484)
Supplement: Attachment — Submitted filename: response_letter_v2.docx [file pcbi.1009484.s038.docx]

To the Editor,

Please consider the revised manuscript entitled “Experiment level curation of transcriptional regulatory interactions in neurodevelopment” by Chu et al. for publication as a research article in PLoS Computational Biology. We, again, thank the editors and reviewers for a thorough review of our work and the valuable feedback. We have now addressed the additional concerns raised by Dr. Lægreid (Reviewer 2). Please see below for the point-by-point response.

Reviewer #1:

*The authors have addressed all of my comments very thoroughly and I would like to acknowledge them for their efforts. The main value of the work in the availability of experimental and contextual details of the curation is now clearly highlighted in the text and newly added validations and comparisons further strengthened the manuscript.*

We thank the reviewer for the positive feedback.

*Reviewer #2:*

**line 343* It would be good if you refer to ENCODE nomenclature as basis for your definition of proximal (since there are a number of different definitions currently in use)*

We thank the reviewer for the suggestion. We have now cited an ENCODE publication (PMID: 32728249) for using the 2 kb threshold (line 333).

**line 366 and other similar parts of text* In your letter to the editor on the revised manuscript you state that the comparison with the high throughput Pax6 perturbation was «intended to be a demonstration of a potential use case of our resource» (page 2).*

*In the manuscript your statements regarding the motivation and outcome of comparing the DTRI resource with observations in high-throughput screens are somewhat heterogenous and span statements pertaining to assessment of high-throughput screen results («...benchmark high-throughput screens» (line 366), «...high confidence DTRI catalogue for training and validating high-throughput predictions» (line 542)) but also pertaining to qualities related to your DTRI resource (e.g. «...suggest that our tissue annotations are accurate» (line 412))*

*It would be good if you could take another look to see whether clarity and stringency can be improved regarding motivations for comparison and premisses for statements regarding the outcome of the comparisons.*

Evaluation of high-throughput predictions, as exemplified by the comparison with the embryonic brain Pax6 perturbation datasets, is the underlying motivation of this work. Using these comparisons, we demonstrated the utility of having detailed curation of experimental and contextual information for each DTRI. We believe that the statements pertaining to assessment of high-throughput results are consistent with the motivation of this study and the sample analysis. As we clarified in our previous response, we did not intend to validate our resource by comparing it to high-throughput screens. We thank the reviewer for pointing out that the statement regarding annotation accuracy (line 396) is unnecessary. We have now removed it from the manuscript.

**line 418* I suggest to provide a few words specifying biological context regarding the «alternative Pax6 pertubation dataset»*

We have added a statement clarifying that the alternative dataset was generated using the embryonic mouse cortex (line 401).

**line 424* Is it 53 or 52 experiments? (Methods section states 52)*

We regret the mistake. It is 53 experiments. We have now corrected it in the manuscript (line 676).

**line 424/line 689* It should be stated whether «all the TFs with more than 10 curated targets» (line 689 Methods section) were taken from only the current DTRI resource, or from the compiled resource including all external resources. If it is the former (only current), this selection strategy is likely to create a bias in comparison with other DTRI resources because the set of TFs with at least 10 targets is likely to vary between resources.*

The set of TFs was selected based on the number of curated targets in the current resource. This strategy helped to limit the scope of the analysis to a small number of TFs. Since we only computed AUROC values for DTRI resource and TF combinations with more than 10 curated targets, this is unlikely to significantly bias our results. Nonetheless, we appreciate the concern. We have now rerun the analysis using a total of 176 datasets (for 37 TFs) for all DTRI resource and TF combinations where there are at least 10 recorded targets (line 422-427). This slightly increased the number of TFs per DTRI resource. Expectedly, the results have remained largely the same (S22 Fig).

**line 424/line 689* It would be helpful for the reader if some details were given in the Result- or Methods section regarding the biological context of the 52/53 TF perturbation sets (cell line, in vivo, organ/tissue type, type of perturbation)*

KnockTF contains a heterogenous set of tissue and cell types. Biological context was not among our selection criteria of the high-throughput TF perturbation datasets. We included all datasets for any TF that has 3 or more datasets available (line 408). We have now included an additional statement addressing the diversity of biological contexts among the datasets in KnockTF (line 408-).
